# Selective Synthesis and Photoluminescence Study of Pyrazolopyridopyridazine Diones and *N*-Aminopyrazolopyrrolopyridine Diones

**DOI:** 10.3390/molecules25102409

**Published:** 2020-05-21

**Authors:** Ching-Chun Tseng, Cheng-Yen Chung, Shuo-En Tsai, Hiroyuki Takayama, Naoto Uramaru, Chin-Yu Lin, Fung Fuh Wong

**Affiliations:** 1 The Ph.D. Program for Biotech Pharmaceutical Industry, China Medical University, No. 91, Hsueh-Shih Rd., Taichung 40402, Taiwan; u106308201@cmu.edu.tw (C.-C.T.); u100003044@cmu.edu.tw (S.-E.T.); 2 School of Pharmacy, China Medical University, No. 91 Hsueh-Shih Rd., Taichung 40402, Taiwan; u108071001@cmu.edu.tw; 3 Department of Medico Pharmaceutical Science, Nihon Pharmaceutical University, Komuro Inamachi Kita-adachi-gun, Saitama-ken 10281, Japan; h.takayama@nichiyaku.ac.jp; 4 Department of Environmental Science, Nihon Pharmaceutical University, Komuro Inamachi Kita-adachi-gun, Saitama-ken 10281, Japan; uramaru@nichiyaku.ac.jp; 5 Institute of New Drug Development, College of Biopharmaceutical and Food Sciences, China Medical University, No. 91 Hsueh-Shih Rd., Taichung 40402, Taiwan; geant@mail.cmu.edu.tw

**Keywords:** pyrazolopyridopyridazine dione, *N*-aminopyrazolopyrrolopyridine dione, luminol, photoluminescence

## Abstract

The newly designed luminol structures of pyrazolopyridopyridazine diones and *N*-aminopyrazolopyrrolopyridine diones were synthesized from versatile 1,3-diaryfuropyrazolopyridine-6,8-diones, 1,3-diarylpyrazolopyrrolopyridine-6,8-diones, or 1,3-diaryl-7-methylpyrazolopyrrolopyridine-6,8-diones with hydrazine monohydrate. Photoluminescent and solvatofluorism properties containing UV–Vis absorption, emission spectra, and quantum yield (Φ_f_) study of pyrazolopyridopyridazine diones and *N*-aminopyrazolopyrrolopyridine diones were also studied. Generally, most of pyrazolopyrrolopyridine-6,8-diones **6** exhibited the significant fluorescence intensity and the substituent effect when compared with *N*-aminopyrazolopyrrolopyridine diones, particularly for **6c** and **6j** with a *m*-chloro group. Additionally, the fluorescence intensity of **6j** was significantly promoted due to the suitable conjugation conformation. Based on the quantum yield (Φ_f_) study, the value of compound **6j** (0.140) with planar structural skeletal was similar to that of standard luminol (**1**, 0.175).

## 1. Introduction

**Figure 1:**
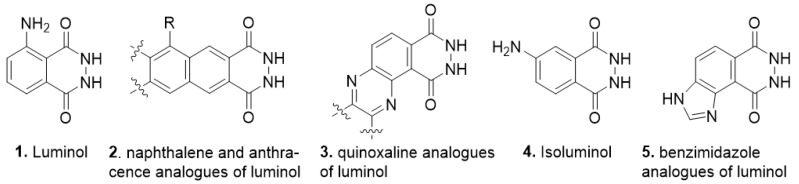
Luminol **1**, naphthalene and anthracene **2**, quinoxaline analogues of luminol **3**, isoluminol **4**, and benzimidazole analogues of luminol **5**.

Sleep-disorders are one of the largest public health concerns in the whole world [1]. New functionalized pyrazolo [3,4-*b*]pyrrolo[3,4-*d*]pyridine derivatives were enthusiastically investigated to develop the increased potency and reduced side effects of novel sedative/hypnotic drug compounds for treatment of sleep-disorders [2,3]. On the other hand, pyrazolopyridopyridazine diones are well-known as the versatile precursors for synthesis of pyrazolopyridopyridazine phosphodiesterase type 5 (PDE5) inhibitors [4,5]. In recent years, chemiluminescent luminol derivatives have been an attractive detection technique in analytical applications such as presumptive test agents for latent blood detection [6,7,8,9], high-performance liquid chromatography (HPLC) [10,11], DNA, immunoassay, and cancer screening detection [12,13]. Since now, many newly designed luminol structures have been enthusiastically investigated to increase the chemiluminescence efficiency, intensity, sensitivity, quantum yield, or the recognition ability of the resulting chemiluminogens (Figure 1) [14,15,16,17,18,19].

**Figure 2:**
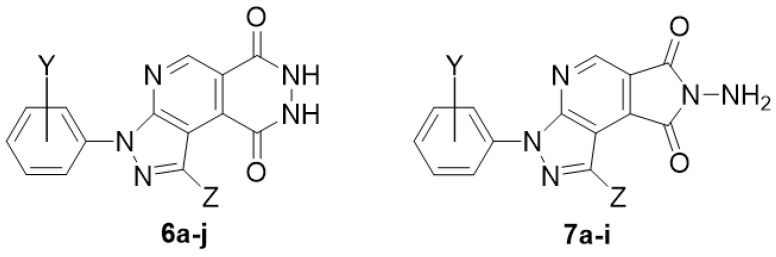
Pyrazolopyridopyridazine diones **6a**–**j** and *N*-Aminopyrazolopyrrolopyridine diones **7a**–**i** as luminol analogues.

Furthermore, *N*-aminophthalimides were considered as phthalazine 1,4-dione tautomeric pairs [20,21]. *N*-Amino maleimides with pyridine heterocycle series also presented as a very important privileged substructure in organic synthesis for preparing diverse biologically active molecules [22]. Typically, the most important pharmacological effects that have been reported are potential antimicrobial [22] and anticancer activities [23]. Herein, we judiciously explore the insertion of pyridazinedione and *N*-Amino maleimide units into the pyrazolopyridine core ring for construction of the new designed luminol structures **6a**–**j** and **7a**–**i** from versatile 1,3-diarylpyrazolopyrrolopyridine-6,8-diones **11**. Observably, we found that the series of pyridazinediones **6a**–**j** would not only provide conjugation systems but also allow to modify the fluorescence intensity and biological activity (Figure 2).

## 2. Results and Discussion

**Scheme 1:**
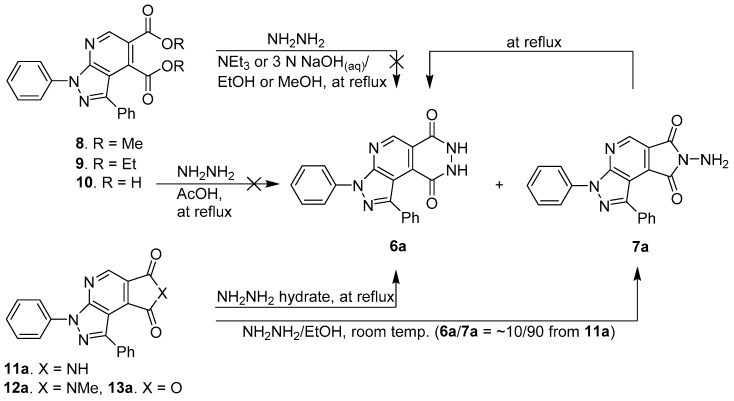
Synthesis study of pyrazolopyridopyridazine dione **6a** as luminol analogue.

Initially, dimethyl 1,3-diphenyl-1*H*-pyrazolo[3,4-*b*]pyridine-4,5-dicarboxylate **8** and diethyl 1,3-diphenyl-1*H*-pyrazolo[3,4-*b*]pyridine-4,5-dicarboxylate **9** were prepared by following our previously reported literature [24] from *N*,*N*-diisopropylamidinyl pyrazolylimine and chosen as the model substrate for this investigation on the construction of pyrazolopyridopyridazine diones **6a** (Scheme 1). Compounds **8** and **9** were reacted with hydrazine hydrate at reflux in methanol or ethanol solution under the basic condition for 24–36 h [25,26]. However, all the efforts for the predominant formation of **6a** were unsuccessful. We also attempted to perform the hydrolysis of ester groups of compounds **8** and **9** under basic conditions to obtain 1,3-diphenyl-1*H*-pyrazolopyridine-4,5-dicarboxylic acid **10 [27,28]**. Subsequently, pyrazolopyridine-4,5-dicarboxylic acid **10** was refluxed with hydrazine in acetic acid to carry out the cyclization for 8 h, but without success (Scheme 1).

In other attempts, we preliminarily tried to synthesize 1,3-phenylpyrazolopyrrolopyridine-6,8-dione **11a** from *N*,*N*-diisopropylamidinyl pyrazolylimine with maleimide via our published InCl_3_/silica gel catalyzed hetero Diels-Alder reaction [29]. Subsequently, the resulting compound **11a** was reacted with an excess of hydrazine hydrate in EtOH/H_2_O co-solution at room temperature for ~7 h [29,30]. The formation of the *N*-aminopyrazolopyrrolopyridine dione **7a** was observed in 83% yield as the major product and accompanied with a trace amount of luminol-type pyrazolopyridopyridazine dione **6a** (<10%, Scheme 1). Fortunately, compounds **6a** and **7a** can be successfully and selectively prepared via kinetic and thermodynamic control reactions [31,32].

For further searching optimal conditions, we also prepared 7-methyl-1,3-phenylpyrazolopyrrolopyridine-6,8-dione **12a** [29] and 1,3-diphenylfuropyrazolopyridine-6,8-dione **13a** [33,34] as probes for monitoring cyclization tendency with hydrazine hydrate [29,30]. Most of the compounds **11a**–**13a** were refluxed in neat hydrazine hydrate solution for ~5 h (Scheme 1 and Table 1). The reactions were monitored until the consumption of starting materials **11a**–**13a** by TLC and produced the luminol-type pyrazolopyridopyridazine dione **6a**. Compound **11a** smoothly underwent the cyclization reaction to give luminol-type analogue **6a** in better yield (84%, Entry 1, Table 1). However, compounds **12a**–**13a** resulted in 32% and 18% low yields, respectively (Entries 2 and 3, Table 1). For further demonstration of reactivity efficiency, compounds **11b**–**c**, **12b**–**c**, and **13b**–**c** bearing various substituents including *o*- and *m*-Cl in *N*-1-phenyl ring and phenyl at *C*-3 position of pyrazole moiety were synthesized and refluxed under the same condition (Entries 4–9, Table 1). Based on the experimental data of Table 1, the better yields of pyrazolopyridopyridazine dione products **6b**–**c** were provided from 1,3-diarylpyrazolopyrrolopyridine-6,8-diones **11b**–**c** (74% and 71%, Entries 4 and 7, Table 1). Unfortunately, 1,3-diaryl-7-methylpyrazolopyrrolopyridine-6,8-diones **12b**–**c** and 1,3-diarylfuropyrazolopyridine-6,8-diones **13b**–**c** showed poor reactivity for the formation of pyrazolopyridopyridazine diones **6b**–**c** (Entries 5–6 and 8–9, Table 1).

Furthermore, we applied this reliable procedure to reactants **11d**–**j** bearing *p*-Cl-Ph, *p*-Br-Ph, *p*-Me-Ph, *p*-OMe-Ph, *p*-CN-Ph, *p*-NO_2_-Ph, and *m*-Cl-Ph at the *N*-1 position and phenyl and H at *C*-3 position of pyrazolic ring. Various substituted reactants **11d**–**j** were demonstrated to proceed smoothly. Both electron-donating and electron-withdrawing substituents were all well-tolerated in good yields (69–84%, Entries 10–16, Table 1). All of 1,3-diarylpyrazolopyrrolopyridine-6,8-diones **6a**–**j** were fully characterized by spectroscopic methods. For example, compound **6a** presented one singlet at *δ* 9.41 ppm for pyrazolopyridine ring N=C*H*–C=C in ^1^H-NMR and two peaks at *δ* 153.1 and 155.7 ppm for pyridazine dione carbon O=C–NH in ^13^C-NMR spectrum. Its IR absorptions showed peaks at 3161 cm^−1^ for stretching of the –NH group and at 1014 cm^−1^ for stretching of the N–N group.

**Scheme 2:**
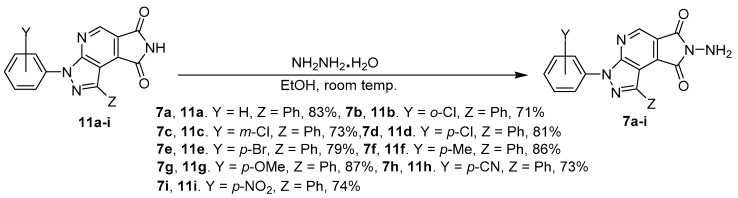
The results of *N*-aminopyrazolopyrrolopyridine diones **7a**–**i** from pyrazolopyrrolopyridine-6,8-diones **11a**–**i** with hydrazine hydrate.

For the further controlled experiment for photoluminescence study, we also tried to prepare a series of *N*-aminopyrazolopyrrolopyridine diones **7a**–**i** as the comparison cases (Scheme 2). Treatment of pyrazolopyrrolopyridine-6,8-diones **11a**–**i** with 5.0 equivalents of hydrazine hydrate in EtOH/H_2_O co-solution was performed in an ice-bath to room temperature for 48 h. The corresponding *N*-aminopyrazolopyrrolopyridine diones **7a**–**i** were obtained in 71–87% yields and characterized by spectroscopic methods. For example, compound **7a** presented one singlet peak at *δ* 8.83 ppm for pyrazolopyridine ring N=C*H*–C=C in ^1^H-NMR and two peaks at *δ* 164.1 and 164.4 ppm for phthalimide moiety carbon O=C–NH in ^13^C-NMR spectrum. Its IR absorptions showed peaks at 3172 and 3276 cm^−1^ for stretching of the –NH_2_ group and at 1014 cm^−1^ for stretching of the N–N group. Due to the structural skeletons being very similar between **6** and **7**, the identify method should be our next future evaluation.

**Figure 3:**
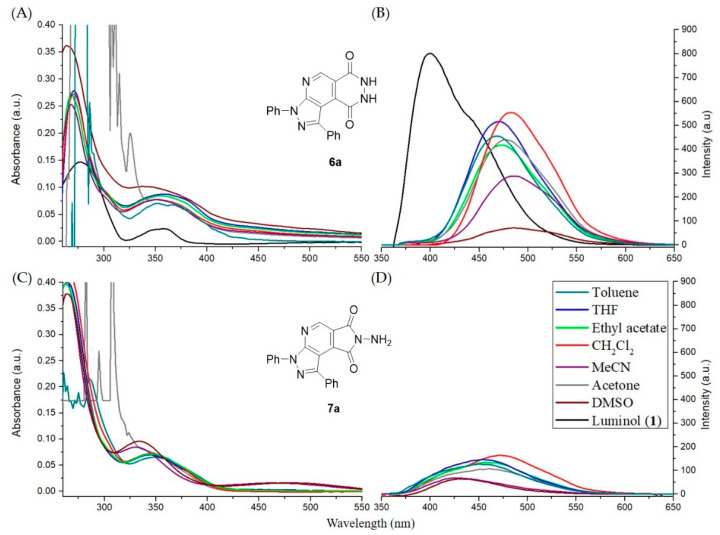
Photoluminescence spectra of luminol (**1**), pyrazolopyridopyridazine dione **6a** and *N*-aminopyrazolopyrrolopyridine dione **7a** in the different solvents. (**A**) Absorption and (**B**) emission spectra of luminol (**1**) and compound **6a**. (**C**) Absorption and (**D**) emission spectra of compound **7a**.

Luminol (**1**), compounds **6a** and **7a** were dissolved in DMSO to prepare a stock solution (1 × 10^−3^ M). Then the stock solutions of compounds **6a** and **7a** were individually diluted to a concentration of 10 μM in the presence of various solvents such as toluene, THF, ethyl acetate (EA), CH_2_Cl_2_, MeCN, acetone, and DMSO. The standard stock solution of luminol (10 μM) was diluted in DMSO solution as the standard sample. The UV–Vis absorption and fluorescence emission spectra of the pyrazolopyridopyridazine dione **6a** and *N*-aminopyrazolopyrrolopyridine dione **7a** compounds in the above-mentioned solution of varying polarities were reported in Table 2. The pyrazolopyridopyridazine dione **6a** has better solubility in polar organic solvents, such as DMSO > THF > acetone, but *N*-aminopyrazolopyrrolopyridine dione **7a** has the solubility only in highly polar solvents like DMSO. Luminol (**1**) was also measured and used as the standard sample. The UV–Vis absorption spectra of the compounds **6a** and **7a** in all the studied solvents were almost nearly the same; their absorption property is independent of the solvent polarity (Figure 3 and Table 2). All these compounds exhibit two highly intense absorption maxima peaks. Among these two, the first one was a high energy absorption between 253 nm and 286 nm for **6a** and **7a** probably due to the π−π* transition of the aryl core [35] while the low energy band between 329 nm and 366 nm is attributed to the intramolecular charge transfer transition (ICT). However, the rigidity in the structure of compounds **6a** and **7a** exhibited the stronger blue-shifted absorption (~15 nm) than luminol (**1**) in DMSO solution, as shown in Figure 3 and Table 2. In comparison with **6a** and **7a**, they demonstrated a similar absorption intensity, and compound **6a** has obvious red-shift ~20 nm with respect to **7a**.

**Figure 4:**
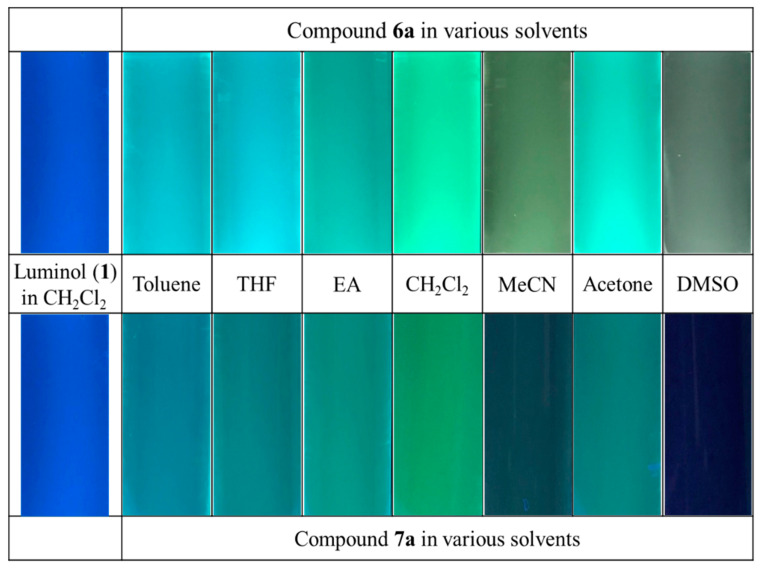
Color pictures of the fluorescence of compounds **6a** and **7a** in various solvents under excitation at 365 nm.

Consequently, we investigated the photoluminescence properties of the compounds **6a** and **7a** with luminol (**1**). For the fluorescence spectra, as shown in Figure 3 and Table 2, both the fluorescence intensity and the maximal position slightly varied depending on the solvent. Compound **6a** displayed a characteristic emission band of the excitation wavelengths between 400 and 600 nm, and the λ_max_s of PL was ~480 nm with the intense greenish-blue fluorescence in Figure 3 and Figure 4. For compound **7a**, it’s emission spectrum was between 350 and 550 nm, and the λ_max_s of PL was ~450 nm with the intense bluish-green fluorescence in Figure 3 and Figure 4. Compounds **6a** and **7a** exhibited a red-shift ~80 nm or ~60 nm as compared to luminol (**1**). Therefore, new luminol analogues **6a** and **7a** were efficiently conjugate and connect two chromophores (pyrazole and pyridine) to lead to an increase of aromaticity and provide the greenish-blue or bluish-green fluorescent materials (Table 2 and Figure 4) [36]. Particularly, the best positive solvatofluorism phenomenon was presented in CH_2_Cl_2_ solution. It was also beneficial for the visibility of the naked eye due to the bathochromic (red-shift) phenomenon from blue color to green (Figure 4).

Moreover, the maximum of fluorescence wavelength and intensity, as shown in Figure 3, significantly vary with the diluted solvent. Further, we surprisingly observed the more significant solvent effect on compound **6a** when compared with compound **7a**. As shown in Figure 3, similar fluorescence spectra but a significant difference in intensity (∼6 times) were observed in varying solvents. Of note, it was interesting that toluene, THF, EA, and CH_2_Cl_2_ had differences in their polarity (toluene: 0.099, THF: 0.207, EA: 0.228, CH_2_Cl_2_: 0.309, with respect to the reference polarity of DMSO: 0.444) [37,38]. However, for the above solvents, we observed a strong intensity, in comparison to that for protic or/and polar solvent (DMSO). The intensities of fluorescence bands were reversed in protic or/and polar solvents. Therefore, the solvent polarity modulation of fluorescence was quite interesting. It was well studied that amide tautomer of pyrazolopyridopyridazine dione **6a** was efficiently produced in toluene, THF, EA, and CH_2_Cl_2_ solvents [39,40,41,42,43,44]. In alcoholic (protic) and DMSO solvent, there exists competition between intermolecular bonding of the nearest hydrogen with the hydroxyimine tautomer of 6-hydroxypyrazolopyridopyridazin-9-one **6a**. Therefore, different intensities of behavior were observed in different polarity solutions. On the other hand, the different fluorescence intensity between structural isomers **6a** and **7a** was also observed [45]. The aromaticity of compound **6a** possessed the bathochromic shift of fluorescence maximum λ_max_ by 12 nm and ∼4 times significant intensity in CH_2_Cl_2_ solution when compared with compound **7a** (Table 2 and Figure 3) [46]. However, the intramolecular and intermolecular hydrogen bondings between the amino and carbonyl groups of *N*-aminopyrazolopyrrolopyridine dione **7a** were formed to lead to the poor intensity in solution.

**Figure 5:**
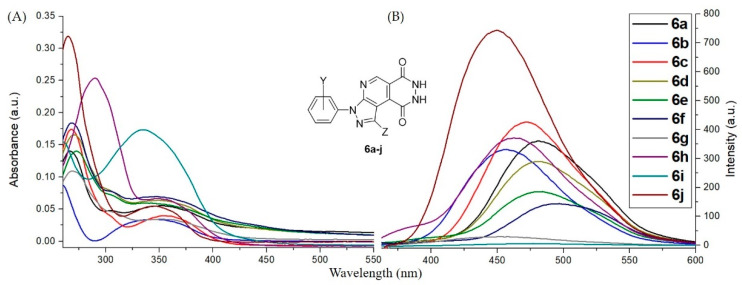
Photoluminescence spectra of pyrazolopyridopyridazine diones **6a**–**j** dissolved in DMSO to prepare a stock solution (1.0 mM). Then the stock solutions were diluted with CH_2_Cl_2_ to a concentration of 10 μM. (**A**) Absorption and (**B**) emission spectra of compounds **6a**–**j**.

**Figure 6:**
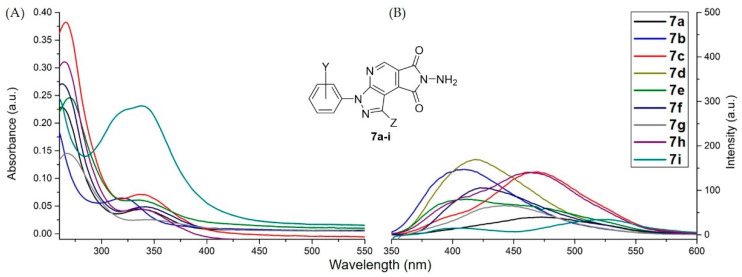
Photoluminescence spectra of *N*-aminopyrazolopyrrolopyridine diones **7a**–**i** dissolved in DMSO to prepare a stock solution (1.0 mM). Then the stock solutions were diluted with CH_2_Cl_2_ to a concentration of 10 μM. (**A**) Absorption and (**B**) emission spectra of compounds **7a**–**i**.

For further investigation of substituent efficiency of compounds **6** and **7** in photoluminescence properties, we synthesized a series of pyrazolopyridopyridazine diones **6a**–**j** and *N*-aminopyrazolopyrrolopyridine diones **7a**–**i** bearing various substituents including *o*-, *m*- and *p*-Cl, *p*-Br, *p*-Me, *p*-OMe, *p*-CN, and *p*-NO_2_ groups in *N*1-phenyl ring of pyrazole moiety. Generally, most of the substituents such as *o*-, *m*- and *p*-Cl, *p*-Br, *p*-Me, and *p*-CN in *N*1-phenyl of pyrazolic ring of compounds **6** possessed the blue-shift phenomenon range ~10 to 30 nm with significant fluorescence intensity when compared with compound **6a**, particularly for **6c** with *meta*-chloro group (Figure 5). For compounds **6g** and **6i** with the strong electron-donating (*p*-OMe) or electron-withdrawing groups (*p*-NO_2_), they exhibited negative photoluminescence properties (Figure 5). While we modified the skeletal structure of pyrazolopyridopyridazine dione **6j**, in which Ph-group was replaced to H atom on *C*-3 position of pyrazolic ring, the blue-shift phenomenon was remarkably observed in photoluminescence spectra. Additionally, the fluorescence intensity of **6j** was significantly promoted about 2.3 times in comparison with compound **6a** (Figure 5). Based on the result of the substituent study, we conceived that compound **6j** was an effective substrate that possessed suitable conjugation conformation without the torsion effect to facilitate the photoluminescence properties [26]. For compounds **7a**–**i** bearing the above various substituents, they provided the weak fluorescence intensity [45] and possessed the blue-shift phenomenon when compared with **7a**, except for **7c** with *m*-chloro group and **7h** with *p*-CN group (Figure 6). Generally, compounds **7a**–**i** were the inappropriate photoluminescent substrates [45].

**Figure 7:**
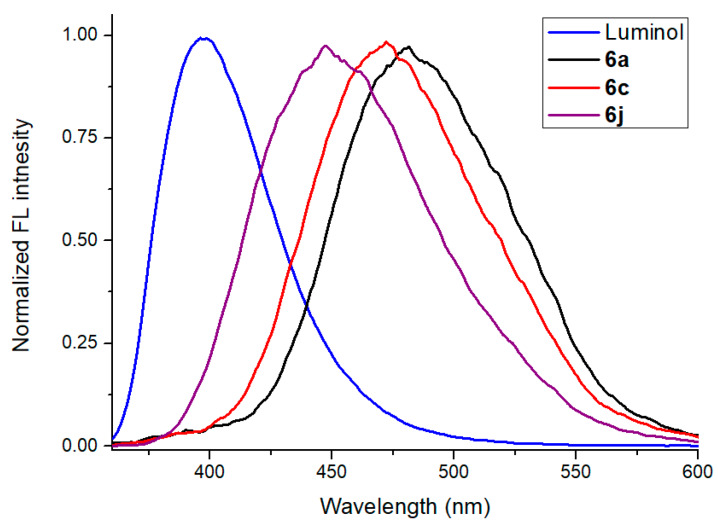
Normalized fluorescence spectra of luminol and pyrazolopyridopyridazine diones **6a**, **6c**, and **6j** in the CH_2_Cl_2_ solution (excitation wavelength 350 nm).

The quantum yields (Φ_f_) of luminol (**1**) and pyrazolopyridopyridazine diones **6a**, **6c**, and **6j** were measured in the CH_2_Cl_2_ solution using quinine sulfate in 0.05M H_2_SO_4_ (Φ_f_ = 0.60) as the standard (excitation wavelength 350 nm) [47,48]. The quantum yields (Φ_f_) values of luminol (**1**) and pyrazolopyridopyridazine diones **6a**, **6c**, and **6j** were estimated as 0.175, 0.056, 0.067, and 0.140 in CH_2_Cl_2_ solution, respectively, indicating that the Φ_f_ value of **6j** was similar to that of luminol (**1**, Table 3). Moreover, we also investigated the quantum yields of **6j** in various solvents by using the same condition. The estimated values order trendy was as 0.218 (THF) > 0.209 (Toluene) > 0.140 (CH_2_Cl_2_) > 0.083 (acetone) > 0.049 (EA), indicating THF provided the largest Φ_f_ value among them (Table 3). On the other hand, most of the quantum yields (Φ_f_) pyrazolopyridopyridazine diones **6a**–**i** in CH_2_Cl_2_ solution were predicted to be an almost identical value (ca. 0.05–0.06). Interestingly, the high Φ_f_ value of **6j** was obtained and possibly caused by a particular improvement in the planar skeletal conformation (Table 3 and Figure 7).

## 3. Experimental Section

### 3.1. General Information

All reagents were used as obtained commercially. All reactions were carried out under argon or nitrogen atmosphere and monitored by thin-layer chromatography (TLC). Flash column chromatography was carried out on silica gel (230–400 mesh). Analytical thin-layer chromatography was performed using pre-coated plates (silica gel 60 F-254) purchased from Merck Inc. Flash column chromatography purification was carried out by gradient elution using *n*-hexane in ethyl acetate (EtOAc) unless otherwise stated. ^1^H-NMR was recorded at 400, 500, or 600 MHz and ^13^C-NMR recorded at 100, 125, or 150 MHz, respectively, in DMSO-*d*_6_ as the solvent. The standard abbreviations s, d, t, q, and m refer to the singlet, doublet, triplet, quartet, and multiplet, respectively. Coupling constant (*J*), whenever discernible, have been reported in Hz. Infrared spectra (IR) were recorded as neat solutions or solids; mass spectra were recorded using electron impact or electrospray ionization techniques. The wavenumbers reported are referenced to the polystyrene 1601 cm^−1^ absorption. ESI-MS analyses were performed on an Applied Biosystems API 300 mass spectrometer. High-resolution mass spectra (HRMS) were recorded on a JEOL JMS-HX110 mass spectrometer with an electron ionization (EI) source The UV-visible absorption and emission spectra were performed on a Perkin-Elmer Lambda 265 and Perkin-Elmer LS50B, a fused quartz cuvette (10 mm × 10 mm) at room temperature, respectively. Quantum yields were obtained by using quinine sulfate (0.60 in 0.05 M H_2_SO_4_) as a reference. Stock solutions (1 × 10^−3^ M) of luminol (**1**), compounds of **6a**–**j** and **7a**–**i** were prepared in dimethyl sulfoxide (DMSO).

### 3.2. Standard Procedure for Synthesis of Pyrazolopyridopyridazine Diones ***6a***–***j***

The reliable procedure involved the treatment of 1,3-diarylpyrazolopyrrolopyridine-6,8-diones (**11a**–**j**), 1,3-diaryl-7-methylpyrazolopyrrolopyridine-6,8-diones (**12a**–**c**) 1,3-diarylfuropyrazolopyridine-6,8-diones (**13a**–**c**, 1.0 equiv.) with hydrazine monohydrate (~40 equiv.) in neat solution at reflux for 5 h. When the reaction was completed, the reaction mixture was added to water (10 mL) for precipitation. The precipitate was filtered, washed with cold water (10 mL), and *n*-hexane/EA (1/2, 15 mL) to give the corresponding crude pyrazolopyridopyridazine diones **6a**–**j**. The crude desired products **6a**–**j** were recrystallized in acetone/THF (1/4) solution to obtain the pure pyrazolopyridopyridazine diones **6a**–**j** in 11–84% yields. The low solubility of the compounds **6a**–**j** made the ^13^C-NMR characterization of quaternary and carbonyl carbons of these substrates unclear [25,26].

*1,3-Diphenyl-7,8-dihydro-3H-pyrazolo[4′,3′:5,6]pyrido[3,4-d]pyridazine-6,9-dione* (**6a**), Light yellow solid; yield: 84%; mp 292–295 °C. ^1^H-NMR (DMSO-*d*_6_, 600 MHz) δ 7.43–7.47 (m, 4H, Ar*H*), 7.60–7.64 (m, 4H, Ar*H*), 8.20 (d, *J* = 7.9 Hz, 2H, Ar*H*), 9.43 (s, 1H, Ar*H*), 10.20 (br, 1H, N*H*); ^13^C{^1^H} NMR (DMSO-*d*_6_, 150 MHz) δ 109.55, 122.46 (2 × CH), 126.67, 127.16, 127.78, 129.26 (2 × CH + 2 × CH), 130.21 (2 × CH + CH), 134.99, 138.16, 147.54, 149.49, 151.35, 153.28, 155.21; FT-IR (KBr) *v*: 3161, 3033, 2907, 1662, 1584, 1499, 1414, 1356, 1306, 906 cm^−1^; MS (EI) *m*/*z* (relative intensity): 356 (24), 355 (M^+^, 100), 354 (27), 270 (24), 269 (12), 268 (12), 77 (39); HRMS (EI) *m*/*z*: [M]^+^ Calcd for C_20_H_13_N_5_O_2_: 355.1069; found: 355.1065.

*3-(2-Chlorophenyl)-1-phenyl-7,8-dihydro-3H-pyrazolo[4′,3′:5,6]pyrido[3,4-d]pyridazine-6,9-dione* (**6b**), Yellow-brown solid; yield: 74%; mp 332–335 °C; ^1^H-NMR (DMSO-*d*_6_, 500 MHz) δ 7.40 (br, 3H, Ar*H*), 7.60–7.61 (m, 2H, Ar*H*), 7.63 (d, *J* = 7.5 Hz, 1H, Ar*H*), 7.67 (t, *J* = 7.5 Hz, 1H, Ar*H*), 7.79–7.80 (m, 2H, Ar*H*), 9.35 (s, 1H, Ar*H*); ^13^C{^1^H} NMR (DMSO-*d*_6_, 125 MHz) δ 109.33, 124.53, 126.66, 127.77, 128.01, 128.18, 128.45, 130.23 (2 × CH), 130.30, 130.47, 131.33, 131.36, 134.73, 134.94, 147.84, 149.81, 152.65, 155.49, 156.94; FT-IR (KBr) *v*: 3427, 3281, 3060, 2921, 1621, 1561, 1508, 1430, 1351, 905 cm^−1^; MS (EI) *m*/*z* (relative intensity): 391 (M^+^ + 2, 29), 390 (22), 389 (M^+^, 100), 355 (12), 354 (52), 304 (15), 268 (17), 111 (13), 77 (44); HRMS (EI) *m*/*z*: [M]^+^ Calcd for C_20_H_12_ClN_5_O_2_: 389.0680; found: 389.0678.

*3-(3-Chlorophenyl)-1-phenyl-7,8-dihydro-3H-pyrazolo[4′,3′:5,6]pyrido[3,4-d]pyridazine-6,9-dione* (**6c**), Yellow solid; yield: 71%; mp 228–229 °C; ^1^H-NMR (DMSO-*d*_6_, 500 MHz) δ 7.42–7.43 (m, 3H, Ar*H*), 7.50 (d, *J* = 6.7 Hz, 1H, Ar*H*), 7.60 (d, *J* = 5.2 Hz, 2H, Ar*H*), 7.64 (t, *J* = 8.0 Hz, 1H, Ar*H*), 8.26 (d, *J* = 8.0 Hz, 1H, Ar*H*), 8.34 (s, 1H, Ar*H*), 9.44 (s, 1H, Ar*H*), 12.06 (br, 1H, N*H*); ^13^C{^1^H} NMR (DMSO-*d*_6_, 125 MHz) δ 107.59, 119.56, 120.40, 121.43, 126.72 (3 × CH), 127.93, 130.14 (2 × CH + 1 × C), 130.99, 133.49, 134.67, 139.34, 148.03, 149.66, 151.53, 155.16, 157.57; FT-IR (KBr) *v*: 3453, 3344, 3296, 1651, 1595, 1483 cm^−1^; MS (EI) *m*/*z* (relative intensity): 391 (M^+^ + 2, 33), 390 (27), 389 (M^+^, 100), 388 (14), 304 (14), 111 (11), 77 (17); HRMS (EI) *m*/*z*: [M]^+^ Calcd for C_20_H_12_ClN_5_O_2_: 389.0680; found: 389.0686.

*3-(4-Chlorophenyl)-1-phenyl-7,8-dihydro-3H-pyrazolo[4′,3′:5,6]pyrido[3,4-d]pyridazine-6,9-dione* (**6d**), Light yellow solid; yield: 81%; mp 339–341 °C; ^1^H-NMR (DMSO-*d*_6_, 500 MHz) δ 7.40–7.41 (m, 3H, Ar*H*), 7.58–7.60 (m, 2H, Ar*H*), 7.68 (d, *J* = 8.9 Hz, 2H, Ar*H*), 8.29 (d, *J* = 8.9 Hz, 2H, Ar*H*), 9.41 (s, 1H, Ar*H*); ^13^C{^1^H} NMR (DMSO-*d*_6_, 125 MHz) δ 107.56, 123.52 (2 × CH), 126.60 (2 × CH), 126.64, 127.77, 127.82, 129.23 (2 × CH), 130.18 (2 × CH), 131.06, 134.89, 137.12, 147.88, 149.80, 151.35, 156.76, 157.30; FT-IR (KBr) *v*: 3345, 3206, 1656, 1494, 1446, 1307, 1094, 902 cm^−1^; MS (EI) *m*/*z* (relative intensity): 391 (M^+^ + 2, 36), 390 (31), 389 (M^+^, 100), 388 (20), 354 (12), 304 (19), 268 (11), 111 (15), 77 (24); HRMS (EI) *m*/*z*: [M]^+^ Calcd for C_20_H_12_ClN_5_O_2_: 389.0680; found: 389.0687.

*3-(4-Bromophenyl)-1-phenyl-7,8-dihydro-3H-pyrazolo[4′,3′:5,6]pyrido[3,4-d]pyridazine-6,9-dione* (**6e**), Light yellow solid; yield: 77%;mp 337–339 °C; ^1^H-NMR (DMSO-*d*_6_, 500 MHz) δ 7.40–7.41 (m, 3H, Ar*H*), 7.59 (d, *J* = 5.5 Hz, 2H, Ar*H*), 7.82 (d, *J* = 9.0 Hz, 2H, Ar*H*), 8.25 (d, *J* = 9.0 Hz, 2H, Ar*H*), 9.42 (s, 1H, Ar*H*); ^13^C{^1^H} NMR (DMSO-*d*_6_, 125 MHz) δ 107.67, 119.42, 123.81 (2 × CH), 126.64 (2 × CH), 127.82, 130.16 (2 × CH + C), 132.16 (2 × CH + C), 134.88, 137.56, 147.92, 149.78, 151.36, 157.59, 159.25; FT-IR (KBr) *v*: 3435, 3345, 3266, 1655, 1536, 1492, 1443, 1307, 1094, 916, 902 cm^−1^; MS (EI) *m*/*z* (relative intensity): 436 (24), 435 (M^+^ + 2, 98), 434 (39), 433 (M^+^, 100), 432 (14), 354 (11), 350 (11), 348 (12), 268 (14), 77 (26); HRMS (EI) *m*/*z*: [M]^+^ Calcd for C_20_H_12_BrN_5_O_2_: 433.0174; found: 433.0171.

*1-Phenyl-3-(p-tolyl)-7,8-dihydro-3H-pyrazolo[4′,3′:5,6]pyrido[3,4-d]pyridazine-6,9-dione* (**6f**), Light yellow solid; yield: 84%; mp 346–348 °C; ^1^H-NMR (DMSO-*d*_6_, 500 MHz) δ 2.40 (s, 3H, C*H*_3_), 7.39–7.42 (m, 5H, Ar*H*), 7.58–7.60 (m, 2H, Ar*H*), 8.07 (d, *J* = 8.4 Hz, 2H, Ar*H*), 9.39 (s, 1H, Ar*H*); ^13^C{^1^H} NMR (DMSO-*d*_6_, 125 MHz) δ 20.61, 106.97, 118.99, 122.32 (2 × CH), 126.60 (2 × CH), 127.68, 128.86, 129.60 (2 × CH), 130.21 (2 × CH), 135.09, 135.83, 136.55, 147.26, 149.35, 151.18, 152.87, 155.65; FT-IR (KBr) *v*: 3436, 3345, 3206, 2919, 1656, 1534, 1514, 1480, 1453, 1310, 1096, 903 cm^−1^; MS (EI) *m*/*z* (relative intensity): 370 (25), 369 (M^+^, 100), 368 (16), 354 (14), 284 (18), 91 (15), 77 (19); HRMS (EI) *m*/*z*: [M]^+^ Calcd for C_21_H_15_N_5_O_2_: 369.1226; found: 369.1216.

*3-(4-Methoxyphenyl)-1-phenyl-7,8-dihydro-3H-pyrazolo[4′,3′:5,6]pyrido[3,4-d]pyridazine-6,9-dione* (**6g**), Deep yellow solid; yield: 81%; mp 311–313 °C; ^1^H-NMR (DMSO-*d*_6_, 500 MHz) δ 3.85 (s, 3H, OC*H*_3_), 7.17 (d, *J* = 11.2 Hz, 2H, Ar*H*), 7.43 (s, 3H, Ar*H*), 7.58–7.60 (m, 2H, Ar*H*), 8.03 (d, *J* = 11.2 Hz, 2H, Ar*H*), 9.38 (s, 1H, Ar*H*); ^13^C{^1^H} NMR (DMSO-*d*_6_, 125 MHz) δ 55.49, 106.64, 114.38 (2 × CH), 115.59, 124.31 (2 × CH), 124.60, 126.71 (2 × CH), 127.77, 130.23 (2 × CH), 131.18, 135.03, 147.01, 149.17, 151.12, 152.63, 156.80, 158.33; FT-IR (KBr) *v*: 3435, 3226, 3065, 2886, 1650, 1590, 1535, 1516, 1441, 1362, 1252, 1170, 905 cm^−1^; MS (EI) *m*/*z* (relative intensity): 386 (24), 385 (M^+^, 100), 370 (13), 77 (18); HRMS (EI) *m*/*z*: [M]^+^ Calcd for C_21_H_15_N_5_O_3_: 385.1175; found: 385.1180.

*3-(4-Cyanophenyl)-1-phenyl-7,8-dihydro-3H-pyrazolo[4′,3′:5,6]pyrido[3,4-d]pyridazine-6,9-dione* (**6h**), Yellow solid; yield: 73%; mp 344–347 °C; ^1^H-NMR (DMSO-*d*_6_, 500 MHz) δ 7.43–7.44 (m, 3H, Ar*H*), 7.60–7.61 (m, 2H, Ar*H*), 8.08 (d, *J* = 6.6 Hz, 2H, Ar*H*), 8.56 (d, *J* = 6.6 Hz, 2H, Ar*H*), 9.45 (s, 1H, Ar*H*); ^13^C{^1^H} NMR (DMSO-*d*_6_, 125 MHz) δ 108.84, 118.51, 121.39, 121.70 (2 × CH), 126.74 (2 × CH), 128.07, 128.63, 130.08 (2 × CH), 130.15, 133.62 (2 × CH), 134.51, 141.67, 148.75, 149.75, 151.87, 155.15, 156.71; FT-IR (KBr) *v*: 3397, 3284, 3056, 2228, 1606, 1569, 1516, 1430, 1400, 1317, 905 cm^−1^; MS (EI) *m*/*z* (relative intensity): 381 (26), 380 (M^+^, 100), 379 (23), 295 (17), 102 (13), 77(29); HRMS (EI) *m*/*z*: [M]^+^ Calcd for C_21_H_12_N_6_O_2_: 380.1022; found: 380.1030.

*3-(4-Nitrophenyl)-1-phenyl-7,8-dihydro-3H-pyrazolo[4′,3′:5,6]pyrido[3,4-d]pyridazine-6,9-dione* (**6i**), Yellow solid; yield: 69%; mp 340–342 °C; ^1^H-NMR (DMSO-*d*_6_, 500 MHz) δ 7.41–7.44 (m, 3H, Ar*H*), 7.61 (d, *J* = 6.3 Hz, 2H, Ar*H*), 8.48 (d, *J* = 8.3 Hz, 2H, Ar*H*), 8.67 (d, *J* = 8.3 Hz, 2H, Ar*H*), 9.47 (s, 1H, Ar*H*), 12.11 (br, 1H, N*H*); ^13^C{^1^H} NMR (DMSO-*d*_6_, 125 MHz) δ 113.65, 121.61 (2 × CH), 124.52, 125.13 (2 × CH), 126.85 (2 × CH), 128.22, 130.12 (2 × CH), 130.27, 134.47, 143.27, 145.04, 149.17, 149.92, 152.09, 154.07, 158.18; FT-IR (KBr) *v*: 3435, 1637, 1596, 1522, 1341, 1112, 905 cm^−1^; MS (EI) *m*/*z*(relative intensity): 401 (23), 400 (M^+^, 100), 370 (22), 315 (11), 77(19); HRMS (EI) *m*/*z*: [M]^+^ Calcd for For C_20_H_12_N_6_O_4_: 400.0920; found: 400.0919.

*3-(3-Chlorophenyl)-7,8-dihydro-3H-pyrazolo[4′,3′:5,6]pyrido[3,4-d]pyridazine-6,9-dione* (**6j**), Light yellow solid; yield: 71%; mp 351–352 °C; ^1^H-NMR (DMSO-*d*_6_, 500 MHz) δ 7.51 (d, *J* = 8.03 Hz, 1H, Ar*H*), 7.66 (t, *J* = 8.0 Hz, 1H, Ar*H*), 8.28 (d, *J* = 8.0 Hz, 1H, Ar*H*), 8.39 (s, 1H, Ar*H*), 8.88 (s, 1H, Ar*H*), 9.40 (s, 1H, Ar*H*), 10.20 (br, 1H, N*H*); ^13^C{^1^H} NMR (DMSO-*d*_6_, 125 MHz) δ 109.53, 119.80, 120.84, 126.68 (2 × C), 131.14 (CH + C), 133.57, 136.04, 139.62, 149.36, 150.78, 152.47, 155.87; FT-IR (KBr) *v*: 3433, 3294, 3168, 2974, 1639, 1594, 1568, 1487, 1448, 1274, 1218, 1125 cm^−1^; MS (EI) *m*/*z* (relative intensity): 315 (M^+^ + 2, 35), 314 (28), 313 (M^+^, 100), 278 (12), 255 (13), 227 (21), 111(12), 75 (11); HRMS (EI) *m*/*z*: [M]^+^ Calcd for C_14_H_8_ClN_5_O_2_: 313.0367; found: 313.0367.

### 3.3. Standard Procedure for Synthesis of N-Aminopyrazolopyrrolopyridine Diones (***7a***–***i***)

The reliable procedure involved the treatment of 1,3-diarylpyrazolopyrrolopyridine-6,8-diones (**11a**–**i**, 1.0 equiv.) with hydrazine monohydrate (~5.0 equiv.) in EtOH/H_2_O (2.0 mL/2.0 mL) in ice-bath to room temperature within 48 h. When the reaction was completed, the reaction mixture was added to water (10 mL) for precipitation. The precipitate was filtered, washed with cold water (10 mL) and *n*-hexane/EA (1/2, 15 mL) to give the corresponding crude *N*-aminophthalimides **7a**–**i**. The crude desired products **7a**–**i** were recrystallized in acetone/THF (1/4) solution to obtain the pure *N*-aminophthalimides **7a**–**i** in 71–87 % yields [31,32].

*7-Amino-1,3-diphenylpyrazolo[3,4-b]pyrrolo[3,4-d]pyridine-6,8-(3H,7H)-dione* (**7a**), White solid; yield: 83%; mp 217–219 °C; ^1^H-NMR (DMSO-*d*_6_, 400 MHz) δ 4.55 (br, 2H, N*H*_2_), 7.42 (t, *J* = 7.5 Hz, 1H, Ar*H*), 7.49–7.55 (m, 3H, Ar*H*), 7.60–7.65 (m, 4H, Ar*H*), 8.25 (d, *J* = 8.1 Hz, 2H, Ar*H*), 8.82 (s, 1 H, Ar*H*); ^13^C{^1^H} NMR (DMSO-*d*_6_, 100 MHz) δ 111.65, 121.43 (2 × CH), 123.37, 126.80, 128.27 (2 × CH), 128.58 (2 × CH), 128.96, 129.41 (2 × CH), 132.04, 138.44, 139.23, 146.02, 148.82, 150.61, 164.18, 164.49; FT-IR (KBr) *v*: 3275, 3208, 3172, 3035, 1633.0, 1572, 1518.7, 1501 cm^−1^; MS (EI) *m*/*z* (relative intensity): 356 (20), 355 (M^+^, 100), 354 (18), 270 (13), 77.0(18); HRMS (EI) *m*/*z*: [M]^+^ Calcd for C_20_H_13_N_5_O_2_: 355.1069; found: 355.1060.

*7-Amino-3-(2-chlorophenyl)-1-phenylpyrazolo[3,4-b]pyrrolo[3,4-d]pyridine-6,8-(3H,7H)-dione* (**7b**), Yellow solid; yield: 71%; mp 165–166 °C; ^1^H-NMR (DMSO-*d*_6_, 500 MHz) δ 4.52 (br, 2H, N*H*_2_), 7.48 (d, *J* = 7.0 Hz, 1H, Ar*H*), 7.52 (d, *J* = 7.1 Hz, 2H, Ar*H*), 7.60–7.68 (m, 4H, Ar*H*), 7.74 (d, *J* = 7.7 Hz, 1H, Ar*H*), 7.79 (d, *J* =7.7 Hz, 1H, Ar*H*), 8.71 (s, 1H, Ar*H*); ^13^C{^1^H} NMR (DMSO-*d*_6_, 125 MHz) δ 108.50, 122.16, 122.88, 123.33, 127.72 (2 × CH), 129.27 (3 × CH), 129.72 (2 × CH), 131.26, 131.46, 136.60, 136.98, 143.86, 145.72, 153.20, 166.81, 168.47; FT-IR (KBr) *v*: 3337, 3296, 2952, 2920, 1778, 1740, 1498, 1375, 1315, 1014 cm^−1^; MS (EI) *m*/*z* (relative intensity): 391 (M^+^ + 2, 32), 390 (22), 389 (M^+^, 100), 355 (18), 354 (90), 304 (12), 268 (11), 77 (18); HRMS (EI) *m*/*z*: [M]^+^ Calcd for C_20_H_12_ClN_5_O_2_: 389.0680; found: 389.0672.

*7-Amino-3-(3-chlorophenyl)-1-phenylpyrazolo[3,4-b]pyrrolo[3,4-d]pyridine-6,8-(3H,7H)-dione* (**7c**), Yellow solid; yield: 73%; mp 173–175 °C; ^1^H-NMR (DMSO-*d*_6_, 400 MHz) δ 4.55 (br, 2H, N*H*_2_), 7.47–7.54 (m, 4H, ArH), 7.64–7.67 (m, 3H, ArH), 8.31 (d, *J* = 8.9 Hz, 1H, Ar*H*), 8.42 (s, 1H, Ar*H*), 8.86 (s, 1H, Ar*H*); ^13^C{^1^H} NMR (DMSO-*d*_6_, 100 MHz) δ 112.05, 119.33, 120.34, 123.72, 126.32, 128.28 (2 × CH), 128.57 (2 × CH), 129.11, 131.21, 131.73, 133.65, 139.41, 139.64, 146.61, 148.98, 150.72, 163.96, 164.33; FT-IR (KBr) *v*: 3264, 3168, 3034, 1649, 1614, 1595, 1488, 1431, 1300, 803 cm^−1^; MS (EI) *m*/*z* (relative intensity): 391 (M^+^ + 2, 33), 390 (24), 389 (M^+^, 100), 374 (13), 304 (11), 77 (14); HRMS (EI) *m*/*z*: [M]^+^ Calcd for C_20_H_12_ClN_5_O_2_: 389.0680; found: 389.0688.

*7-Amino-3-(4-chlorophenyl)-1-phenylpyrazolo[3,4-b]pyrrolo[3,4-d]pyridine-6,8-(3H,7H)-dione* (**7d**), Light yellow solid; yield: 81%; mp 226–227 °C; ^1^H-NMR (DMSO-*d*_6_, 500 MHz) δ 4.55 (br, 2H, N*H*_2_), 7.48–7.55 (m, 3H, Ar*H*), 7.64 (d, *J* = 7.6 Hz, 2H, Ar*H*), 7.69 (d, *J* = 8.4 Hz, 2H, Ar*H*), 8.35 (d, *J* = 8.4 Hz, 2H, Ar*H*), 8.84 (s, 1 H, Ar*H*); ^13^C{^1^H} NMR (DMSO-*d*_6_, 125 MHz) δ 111.85, 122.54 (2 × CH), 123.55, 128.24 (2 × CH), 128.53 (2 × CH), 129.03, 129.36 (2 × CH), 130.67, 131.81, 137.31, 139.34, 146.35, 148.88, 150.57, 163.99, 164.34; FT-IR (KBr) *v*: 3275, 3207, 3170, 1633, 1499, 828 cm^−1^; MS (EI) *m*/*z* (relative intensity): 391 (M^+^ + 2, 34), 390 (28), 389 (M^+^, 100), 388 (15), 304 (12), 77 (13); HRMS (EI) *m*/*z*: [M]^+^ Calcd for C_20_H_12_ClN_5_O_2_: 389.0680; found: 389.0686.

*7-Amino-3-(4-bromophenyl)-1-phenylpyrazolo[3,4-b]pyrrolo[3,4-d]pyridine-6,8-(3H,7H)-dione* (**7e**), Yellow solid; yield: 79%; mp 235–239 °C; ^1^H-NMR (DMSO-*d*_6_, 500 MHz) δ 4.55 (br, 2H, N*H*_2_), 7.49–7.55 (m, 3H, Ar*H*), 7.64 (d, *J* = 6.9 Hz, 2H, Ar*H*), 7.82 (d, *J* = 8.7 Hz, 2H, Ar*H*), 8.29 (d, *J* = 8.7 Hz, 2H, Ar*H*), 8.84 (s, 1 H, Ar*H*); ^13^C{^1^H} NMR (DMSO-*d*_6_, 125 MHz) δ 111.91, 118.95, 122.81 (2 × CH), 123.55, 128.25 (2 × CH), 128.54 (2 × CH), 129.04, 131.80, 132.28 (2 × CH), 137.75, 139.33, 146.40, 148.90, 150.59, 164.00, 164.35; FT-IR (KBr) *v*: 3275, 3208, 3170, 3071, 3037, 1632, 1495, 826 cm^−1^; MS (EI) *m*/*z* (relative intensity): 436 (22), 435 (M^+^ + 2, 100), 354 (33), 433 (M^+^, 99), 432 (12), 420 (19), 419 (11), 418 (19), 354 (11), 350 (10), 348 (11), 268 (17), 77 (26); HRMS (EI) *m*/*z*: [M]^+^ Calcd for C_20_H_12_BrN_5_O_2_: 433.0174; found: 433.0171.

*7-Amino-1-phenyl-3-(p-tolyl)pyrazolo[3,4-b]pyrrolo[3,4-d]pyridine-6,8-(3H,7H)-dione* (**7f**), Yellow solid; yield: 86%; mp 232–233 °C; ^1^H-NMR (DMSO-*d*_6_, 500 MHz) δ 2.40 (s, 3H, C*H*_3_), 4.55 (br, 2H, N*H*_2_), 7.42 (d, *J* = 8.0 Hz, 2H, Ar*H*), 7.49 (d, *J* = 7.3 Hz, 1H, Ar*H*), 7.52 (t, *J* = 7.3 Hz, 2H, Ar*H*), 7.64 (d, *J* = 7.3 Hz, 2H, Ar*H*), 8.12 (d, *J* = 8.0 Hz, 2H Ar*H*), 8.81 (s, 1H, Ar*H*); ^13^C{^1^H} NMR (DMSO-*d*_6_, 125 MHz) δ 20.63, 111.44, 121.35(2 × CH), 123.18, 128.21 (2 × CH), 128.54 (2 × CH), 128.85, 129.73 (2 × CH), 132.09, 136.06, 136.18, 139.13, 145.69, 148.72, 150.44, 164.20, 164.50; FT-IR (KBr) *v*: 3276, 3209, 3171, 3032, 1634, 1517 cm^−1^; MS (EI) *m*/*z* (relative intensity): 370 (25), 369 (M^+^, 100), 368 (13), 354 (21), 284 (12), 207 (10), 91.1(11), 77.1(13); HRMS (EI) *m*/*z*: [M]^+^ Calcd for C_21_H_15_N_5_O_2_: 369.1226; found: 369.1231.

*7-Amino-3-(4-methoxyphenyl)-1-phenylpyrazolo[3,4-b]pyrrolo[3,4-d]pyridine-6,8-(3H,7H)-dione* (**7g**), Yellow solid; yield: 87%; mp 331–332 °C; ^1^H-NMR (DMSO-*d*_6_, 500 MHz) δ 3.83 (s, 3H, OC*H*_3_), 4.53 (br, 2H, N*H*_2_), 7.17 (d, *J* = 8.7 Hz, 2H, Ar*H*), 7.48–7.53 (m, 3H, Ar*H*), 7.62 (d, *J* = 7.1 Hz, 2H, Ar*H*), 8.06 (d, *J* = 8.7 Hz, 2H, Ar*H*), 8.78 (s, 1H, ArH); ^13^C{^1^H} NMR (DMSO-*d*_6_, 125 MHz) δ 55.64, 111.24, 114.62 (2 × CH), 123.14, 123.51 (2 × CH), 128.37 (2 × CH), 128.65 (2 × CH), 128.97, 131.60, 132.25, 139.14, 145.54, 148.83, 150.44, 158.16, 164.43, 164.73; FT-IR (KBr) *v*: 3215, 3066, 3004, 2963, 2935, 2837, 1639, 1577, 1516, 1462, 1443, 1252 cm^−1^; MS (EI) *m*/*z* (relative intensity): 386 (21), 385 (M^+^, 100), 370 (14), 77.0(10); HRMS (EI) *m*/*z*: [M]^+^ Calcd for C_21_H_15_N_5_O_3_: 385.1175; found: 385.1182.

*7-Amino-3-(4-cyanophenyl)-1-phenylpyrazolo[3,4-b]pyrrolo[3,4-d]pyridine-6,8-(3H,7H)-dione* (**7h**), Yellow solid; yield: 73%; mp 333–335 °C; ^1^H-NMR (DMSO-*d*_6_, 500 MHz) δ 4.55 (br, 2H, N*H*_2_), 7.50–7.55 (m, 3H, Ar*H*), 7.65 (d, *J* = 7.1 Hz, 2H, Ar*H*), 8.09 (d, *J* = 8.5 Hz, 2H, Ar*H*), 8.61 (d, *J* = 8.5 Hz, 2H, Ar*H*), 8.87 (s, 1 H, Ar*H*); ^13^C{^1^H} NMR (DMSO-*d*_6_, 125 MHz) δ 108.41, 112.58, 118.68, 120.75 (2 × CH), 124.12, 128.35 (2 × CH), 128.59 (2 × CH), 129.33, 131.55, 133.84 (2 × CH), 139.53, 141.99, 147.47, 149.11, 151.12, 163.83, 164.27; FT-IR (KBr) *v*: 3330, 3274, 2227, 1665, 1635, 1607, 1518, 1409, 1255, 844 cm^−1^; MS (EI) *m*/*z* (relative intensity): 381 (22), 380 (M^+^, 100), 379 (18), 295 (13), 77(14); HRMS (EI) *m*/*z*: [M]^+^ Calcd for C_21_H_12_N_6_O_2_: 380.1022; found: 380.1023.

*7-Amino-3-(4-nitrophenyl)-1-phenylpyrazolo[3,4-b]pyrrolo[3,4-d]pyridine-6,8-(3H,7H)-dione* (**7i**), Yellow solid; yield: 74%; mp 281–282 °C; ^1^H-NMR (DMSO-*d*_6_, 500 MHz) δ 7.53–7.57 (m, 3H, Ar*H*), 7.93 (d, *J* = 6.5 Hz, 2H, Ar*H*), 8.48 (d, *J* = 9.2 Hz, 2H, Ar*H*), 8.67 (d, *J* = 9.2 Hz, 2H, Ar*H*), 9.19 (s, 1H, Ar*H*); ^13^C{^1^H} NMR (DMSO-*d*_6_, 125 MHz) δ 109.57, 121.41 (2 × CH), 123.52, 125.21 (2 × CH), 127.91 (2 × CH), 129.73, 129.90 (2 × CH), 131.21, 136.89, 143.24, 144.26, 145.12, 147.11, 153.90, 166.71, 168.43; FT-IR (KBr) *v*: 3190, 3120, 3064, 1595, 1500, 1341, 1112, 857 cm^−1^; MS (EI) *m*/*z* (relative intensity): 400 (M^+^, 4), 386 (26), 385 (100), 338 (13), 236 (10), 77 (13); HRMS (EI) *m*/*z*: [M]^+^ Calcd for C_20_H_12_N_6_O_4_: 400.0920; found: 400.0925.

### 3.4. Determination of the Fluorescence Quantum Yield

The fluorescence quantum yield Φ_x_ was determined through the comparative method. The quinine sulfate (Φ_st_ = 0.60, λ_ex_= 350 nm) in H_2_SO_4_ 0.05 M was used as the standard, and it was calculated by following equation [48]:

Φ_x_/Φ_st_ = [A_st_/A_x_] [n_x_^2^/n_st_^2^] [D_x_/D_st_], (1)

where st: standard; x: sample; Φ: quantum yield; A: absorbance at the excitation wavelength; D: area under the fluorescence spectra on an energy scale; n: the refractive index of the solution. In the process of detection, the absorbance should be controlled and lower than 0.1.

## 4. Conclusions

Pyrazolopyridopyridazine diones **6** and *N*-aminopyrazolopyrrolopyridine diones **7** can be prepared in three synthesis methods from 1,3-diarylpyrazolopyrrolopyridine-6,8-diones, 1,3-diaryl-7-methylpyrazolopyrrolopyridine-6,8-diones, or 1,3-diarylfuropyrazolopyridine-6,8-diones with hydrazine monohydrate. Based on the experimental results, 1,3-diarylpyrazolopyrrolopyridine-6,8-diones were conceived as the best reactive starting materials. Furthermore, compounds **6** and **7** were also selectively synthesized under kinetic and thermodynamic control reactions. For the further photoluminescence, solvatofluorism, and quantum yield (Φ_f_) studies, pyrazolopyridopyridazine diones **6** generally exhibited the stronger fluorescence intensity and possessed the significant substituent effect, particularly for **6c** with a *m*-chloro group. On the other hand, the best Φ_f_ value of **6j** was obtained (Φ_f_ = 0.140) and similar to luminol (**1**, Φ_f_ = 0.175), possibly caused by the planar skeletal conformation. Based on the above photoluminescence studies, we also found that the efficient introduction of the pyrazole and pyridine chromophores led to an increase in the conjugation and aromaticity of compounds **6** and **7** when compared with the standard luminol.

## Figures and Tables

**Table 1:**
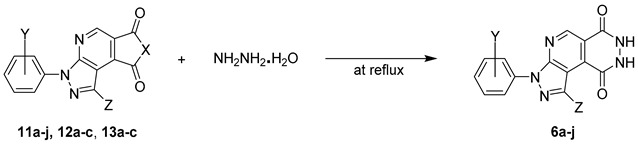
The results of pyrazolopyridopyridazine diones **6a**–**j** from reactants **11a**–**j**, **12a**–**c**, or **13a**–**c** with hydrazine hydrate.

Entry	S.M.	X	Y	Z	Reaction Time	Products	Yields (%)
1	**11a**	NH	Ph	Ph	5	**6a**	84
2	**12a**	NMe	Ph	Ph	5	**6a**	32
3	**13a**	O	Ph	Ph	5	**6a**	18
4	**11b**	NH	*o*-Cl-Ph	Ph	5	**6b**	74
5	**12b**	NMe	*o*-Cl-Ph	Ph	5	**6b**	24
6	**13b**	O	*o*-Cl-Ph	Ph	5	**6b**	13
7	**11c**	NH	*m*-Cl-Ph	Ph	5	**6c**	71
8	**12c**	NMe	*m*-Cl-Ph	Ph	5	**6c**	38
9	**13c**	O	*m*-Cl-Ph	Ph	5	**6c**	11
10	**11d**	NH	*p*-Cl-Ph	Ph	5	**6d**	81
11	**11e**	NH	*p*-Br-Ph	Ph	5	**6e**	77
12	**11f**	NH	*p*-Me-Ph	Ph	5	**6f**	84
13	**11g**	NH	*p*-OMe-Ph	Ph	5	**6g**	81
14	**11h**	NH	*p*-CN-Ph	Ph	5	**6h**	73
15	**11i**	NH	*p*-NO_2_-Ph	Ph	5	**6i**	69
16	**11j**	NH	*m*-Cl-Ph	H	5	**6j**	71

**Table 2:** UV-Vis absorption maximum and fluorescence emission peak wavelength of luminol (**1**), pyrazolopyridopyridazine dione **6a** and *N*-aminopyrazolopyrrolopyridine dione **7a** in the different solvents.

Compound	Solvent	λmax/nm of UV-Vis	λmax/nm of PL
**6a**	Toluene	- ^1^,366	469
**6a**	THF	271,358	471
**6a**	Ethyl acetate	268, 356	473
**6a**	CH_2_Cl_2_	271, 350	483
**6a**	MeCN	268, 351	488
**6a**	Acetone	- ^1^, 353	477
**6a**	DMSO	264, 338	486
**7a**	Toluene	286, ^1^ ,348	452
**7a**	THF	264, 344	454
**7a**	Ethyl acetate	262, 343	459
**7a**	CH_2_Cl_2_	264, 344	471
**7a**	MeCN	261, 329	425, 461
**7a**	Acetone	- ^1^, 338	452
**7a**	DMSO	264, 335	429, 478
Luminol (**1**)	DMSO	350	392

^1^ It was overlapped with solvent absorption band.

**Table 3:** Quantum yields of fluorescence of luminol (**1**) and pyrazolopyridopyridazine diones **6a**, **6c**, and **6j**.

Compound	Solvent	λ_fl_^1^/nm	Φ_f_^2^
**6a**	CH_2_Cl_2_	481	0.056
**6c**	CH_2_Cl_2_	472	0.067
**6j**	CH_2_Cl_2_	450	0.140
**6j**	THF	435	0.218
**6j**	Toluene	438	0.209
**6j**	Acetone	437	0.083
**6j**	Ethyl acetate	437	0.049
Luminol (**1**)	CH_2_Cl_2_	399	0.175

^1^ Fluorescence maximum wavelength (λ_fl_). ^2^ Φ_f_: Fluorescence quantum efficiency, relative to quinine sulfate (Φ_f_ = 0.60).

## Supplementary Materials

The following are available online, copies of ^1^H and ^13^C-NMR spectra of compounds **6a**–**6j** and **7a**–**7i**.
