# Supplementary material for: Selective Synthesis and Photoluminescence Study of Pyrazolopyridopyridazine Diones and N-Aminopyrazolopyrrolopyridine Diones
Source: Molecules. 2020 May 21;25(10):2409. doi: 10.3390/molecules25102409 (PMC7288053; doi:10.3390/molecules25102409)

molecules

Article

Selective synthesis and photoluminescence study of pyrazolopyridopyridazine diones and *N*-Aminopyrazolopyrrolopyridine diones

Ching-Chun Tseng [1,2], Cheng-Yen Chung [2], Shuo-En Tsai [1,2], Hiroyuki Takayama [3], Naoto Uramaru [4], Chin-Yu Lin [5] and Fung Fuh Wong [1,2,*]

1 The Ph.D. Program for Biotech Pharmaceutical Industry, China Medical University, No. 91, Hsueh-Shih Rd., Taichung, 40402, Taiwan; u106308201@cmu.edu.tw (C.-C.T.); u100003044@cmu.edu.tw (S.-E.T.)
2 School of Pharmacy, China Medical University, No. 91 Hsueh-Shih Rd., Taichung 40402, Taiwan; u108071001@cmu.edu.tw (C.-Y.C.)
3 Department of Medico Pharmaceutical Science, Nihon Pharmaceutical University, Komuro Inamachi Kita-adachi-gun, Saitama-ken 10281, Japan; h.takayama@nichiyaku.ac.jp
4 Department of Environmental Science, Nihon Pharmaceutical University, Komuro Inamachi Kita-adachi-gun, Saitama-ken 10281, Japan; uramaru@nichiyaku.ac.jp
5 Institute of New Drug Development, College of Biopharmaceutical and Food Sciences, China Medical University, No. 91 Hsueh-Shih Rd., Taichung 40402, Taiwan; geant@mail.cmu.edu.tw
* Correspondence: wongfungfuh@yahoo.com.tw or ffwong@mail.cmu.edu.tw; Tel.: +886-422-053-366 (ext. 5603); Fax: +886-422-078-083

Supporting information

Table of Contents

1. General Experimental Procedures....................S2

2. NMR Spectra of Reported Compounds....................S6

General Experimental Procedures

1.1. General Information

All reagents were used as obtained commercially. All reactions were carried out under argon or nitrogen atmosphere and monitored by TLC. Flash column chromatography was carried out on silica gel (230–400 mesh). Analytical thin-layer chromatography (TLC) was performed using precoated plates (silica gel 60 F-254) purchased from Merck Inc. Flash column chromatography purification was carried out by gradient elution using *n*-hexane in ethyl acetate (EtOAc) unless otherwise stated. ^{1}H NMR was recorded at 400, 500, or 600 MHz and ^{13}C NMR recorded at 100, 125, or 150 MHz, respectively, in DMSO-d_6 as the solvent. The standard abbreviations s, d, t, q, and m refer to the singlet, doublet, triplet, quartet, and multiplet, respectively. Coupling constant (*J*), whenever discernible, have been reported in Hz. Infrared spectra (IR) were recorded as neat solutions or solids; mass spectra were recorded using electron impact or electrospray ionization techniques. The wavenumbers reported are referenced to the polystyrene 1601 cm^{-1} absorption. ESI-MS analyses were performed on an Applied Biosystems API 300 mass spectrometer. High-resolution mass spectra (HRMS) were recorded on a JEOL JMS-HX110 mass spectrometer with an electron ionization (EI) source The UV-visible absorption and emission spectra were performed on a Perkin-Elmer Lambda 265 and Perkin-Elmer LS50B, a fused quartz cuvette (10 mm × 10 mm) at room temperature, respectively. Quantum yields were obtained by using quinine sulfate (0.60 in 0.05 M H_2SO_4) as a reference. Stock solutions (1 × 10^{-3} M) of Luminol (**1**), compounds of **6a**–**j** and **7a**–**i** were prepared in dimethyl sulfoxide (DMSO).

1.2. Standard procedure for synthesis of pyrazolopyridopyridazine diones 6a–j

The reliable procedure were involved the treatment of 1,3-diarylpyrazolopyrrolopyridine-6,8-diones (**11a–j**), 1,3-diaryl-7-methylpyrazolopyrrolopyridine-6,8-diones (**12a–c**) 1,3-diarylfuropyrazolopyridine-6,8-diones (**13a–c**, 1.0 equiv.) with hydrazine monohydrate (~40 equiv.) in neat solution at reflux for 5 h. When the reaction was completed, the reaction mixture was added to water (10 mL) for precipitation. The precipitate was filtered, washed with cold water (10 mL) and *n*-hexane/EA (1/2, 15 mL) to give the corresponding crude pyrazolopyridopyridazine diones **6a–j**. The crude desired products **6a–j** were recrystallized in acetone/THF (1/4) solution to obtain the pure pyrazolopyridopyridazine diones **6a–j** in 11–84 % yields. The low solubility of the compounds **6a–j** made the ^{13}C NMR characterization of quaternary and carbonyl carbons of these substrates unclear.

1,3-Diphenyl-7,8-dihydro-3H-pyrazolo[4′,3′:5,6]pyrido[3,4-d]pyridazine-6,9-dione (**6a**), Light yellow solid; yield: 84%; mp 292–295 °C. ^{1}H NMR (DMSO-d_6, 600 MHz) δ 7.43–7.47 (m, 4H, Ar*H*), 7.60–7.64 (m, 4H, Ar*H*), 8.20 (d, *J* = 7.9 Hz, 2H, Ar*H*), 9.43 (s, 1H, Ar*H*), 10.20 (br, 1H, N*H*); ^{13}C{^{1}H} NMR (DMSO-d_6, 150 MHz) δ 109.55, 122.46 (2 × CH), 126.67, 127.16, 127.78, 129.26 (2 × CH + 2 × CH), 130.21 (2 × CH + CH), 134.99, 138.16, 147.54, 149.49, 151.35, 153.28, 155.21; FT-IR (KBr) *v*: 3161, 3033, 2907, 1662, 1584, 1499, 1414, 1356, 1306, 906 cm^{-1}; MS (EI) *m*/*z* (relative intensity): 356 (24), 355 (M$^+$, 100), 354 (27), 270 (24), 269 (12), 268 (12), 77 (39); HRMS (EI) m/z: [M]$^+$ Calcd for $C_{20}H_{13}N_5O_2$: 355.1069; found: 355.1065.

3-(2-Chlorophenyl)-1-phenyl-7,8-dihydro-3H-pyrazolo[4′,3′:5,6]pyrido[3,4-d]pyridazine-6,9-dione (**6b**), Yellow-brown solid; yield: 74%; mp 332–335 °C; ^{1}H NMR (DMSO-d_6, 500 MHz) δ 7.40 (br, 3H, Ar*H*), 7.60–7.61 (m, 2H, Ar*H*), 7.63 (d, *J* = 7.5 Hz, 1H, Ar*H*), 7.67 (t, *J* = 7.5 Hz, 1H, Ar*H*), 7.79–7.80 (m, 2H, Ar*H*), 9.35 (s, 1H, Ar*H*); ^{13}C{^{1}H} NMR (DMSO-d_6, 125 MHz) δ 109.33, 124.53, 126.66, 127.77, 128.01, 128.18, 128.45, 130.23 (2 × CH), 130.30, 130.47, 131.33, 131.36, 134.73, 134.94, 147.84, 149.81, 152.65, 155.49, 156.94; FT-IR (KBr) *v*: 3427, 3281, 3060, 2921, 1621, 1561, 1508, 1430, 1351, 905 cm^{-1}; MS (EI) *m*/*z* (relative intensity): 391 (M$^+$ + 2, 29), 390 (22), 389 (M$^+$, 100), 355 (12), 354 (52), 304 (15), 268 (17), 111 (13), 77 (44); HRMS (EI) m/z: [M]$^+$ Calcd for $C_{20}H_{12}ClN_5O_2$: 389.0680; found: 389.0678.

3-(3-Chlorophenyl)-1-phenyl-7,8-dihydro-3H-pyrazolo[4′,3′:5,6]pyrido[3,4-d]pyridazine-6,9-dione (**6c**), Yellow solid; yield: 71%; mp 228–229 °C; ^{1}H NMR (DMSO-d_6, 500 MHz) δ 7.42–7.43 (m, 3H, Ar*H*), 7.50 (d, *J* = 6.7 Hz, 1H, Ar*H*), 7.60 (d, *J* = 5.2 Hz, 2H, Ar*H*), 7.64 (t, *J* = 8.0 Hz, 1H, Ar*H*), 8.26 (d, *J* = 8.0 Hz, 1H, Ar*H*), 8.34 (s, 1H, Ar*H*), 9.44 (s, 1H, Ar*H*), 12.06 (br, 1H, N*H*); ^{13}C{^{1}H} NMR (DMSO-d_6, 125 MHz) δ 107.59, 119.56, 120.40, 121.43, 126.72 (3 × CH), 127.93, 130.14 (2 × CH + 1 × C), 130.99, 133.49,

134.67, 139.34, 148.03, 149.66, 151.53, 155.16, 157.57; FT-IR (KBr) *v*: 3453, 3344, 3296, 1651, 1595, 1483 cm^{-1}; MS (EI) *m*/*z* (relative intensity): 391 (M$^+$ + 2, 33), 390 (27), 389 (M$^+$, 100), 388 (14), 304 (14), 111 (11), 77 (17); HRMS (EI) m/z: [M]$^+$ Calcd for $C_{20}H_{12}ClN_5O_2$: 389.0680; found: 389.0686.

3-(4-Chlorophenyl)-1-phenyl-7,8-dihydro-3H-pyrazolo[4′,3′:5,6]pyrido[3,4-d]pyridazine-6,9-dione (**6d**), Light yellow solid; yield: 81%; mp 339–341 °C; ^{1}H NMR (DMSO-*d*$_6$, 500 MHz) δ 7.40–7.41 (m, 3H, Ar*H*), 7.58–7.60 (m, 2H, Ar*H*), 7.68 (d, *J* = 8.9 Hz, 2H, Ar*H*), 8.29 (d, *J* = 8.9 Hz, 2H, Ar*H*), 9.41 (s, 1H, Ar*H*); ^{13}C{^{1}H} NMR (DMSO-*d*$_6$, 125 MHz) δ 107.56, 123.52 (2 × CH), 126.60 (2 × CH), 126.64, 127.77, 127.82, 129.23 (2 × CH), 130.18 (2 × CH), 131.06, 134.89, 137.12, 147.88, 149.80, 151.35, 156.76, 157.30; FT-IR (KBr) *v*: 3345, 3206, 1656, 1494, 1446, 1307, 1094, 902 cm^{-1}; MS (EI) *m*/*z* (relative intensity): 391 (M$^+$ + 2, 36), 390 (31), 389 (M$^+$, 100), 388 (20), 354 (12), 304 (19), 268 (11), 111 (15), 77 (24); HRMS (EI) m/z: [M]$^+$ Calcd for $C_{20}H_{12}ClN_5O_2$: 389.0680; found: 389.0687.

3-(4-Bromophenyl)-1-phenyl-7,8-dihydro-3H-pyrazolo[4′,3′:5,6]pyrido[3,4-d]pyridazine-6,9-dione (**6e**), Light yellow solid; yield: 77%;mp 337–339 °C; ^{1}H NMR (DMSO-*d*$_6$, 500 MHz) δ 7.40–7.41 (m, 3H, Ar*H*), 7.59 (d, *J* = 5.5 Hz, 2H, Ar*H*), 7.82 (d, *J* = 9.0 Hz, 2H, Ar*H*), 8.25 (d, *J* = 9.0 Hz, 2H, Ar*H*), 9.42 (s, 1H, Ar*H*); ^{13}C{^{1}H} NMR (DMSO-*d*$_6$, 125 MHz) δ 107.67, 119.42, 123.81 (2 × CH), 126.64 (2 × CH), 127.82, 130.16 (2 × CH + C), 132.16 (2 × CH + C), 134.88, 137.56, 147.92, 149.78, 151.36, 157.59, 159.25; FT-IR (KBr) *v*: 3435, 3345, 3266, 1655, 1536, 1492, 1443, 1307, 1094, 916, 902 cm^{-1}; MS (EI) *m*/*z* (relative intensity): 436 (24), 435 (M$^+$ + 2, 98), 434 (39), 433 (M$^+$, 100), 432 (14), 354 (11), 350 (11), 348 (12), 268 (14), 77 (26); HRMS (EI) m/z: [M]$^+$ Calcd for $C_{20}H_{12}BrN_5O_2$: 433.0174; found: 433.0171.

1-Phenyl-3-(p-tolyl)-7,8-dihydro-3H-pyrazolo[4′,3′:5,6]pyrido[3,4-d]pyridazine-6,9-dione (**6f**), Light yellow solid; yield: 84%; mp 346–348 °C; ^{1}H NMR (DMSO-*d*$_6$, 500 MHz) δ 2.40 (s, 3H, C*H*$_3$), 7.39–7.42 (m, 5H, Ar*H*), 7.58–7.60 (m, 2H, Ar*H*), 8.07 (d, *J* = 8.4 Hz, 2H, Ar*H*), 9.39 (s, 1H, Ar*H*); ^{13}C{^{1}H} NMR (DMSO-*d*$_6$, 125 MHz) δ 20.61, 106.97, 118.99, 122.32 (2 × CH), 126.60 (2 × CH), 127.68, 128.86, 129.60 (2 × CH), 130.21 (2 × CH), 135.09, 135.83, 136.55, 147.26, 149.35, 151.18, 152.87, 155.65; FT-IR (KBr) *v*: 3436, 3345, 3206, 2919, 1656, 1534, 1514, 1480, 1453, 1310, 1096, 903 cm^{-1}; MS (EI) *m*/*z* (relative intensity): 370 (25), 369 (M$^+$, 100), 368 (16), 354 (14), 284 (18), 91 (15), 77 (19); HRMS (EI) m/z: [M]$^+$ Calcd for $C_{21}H_{15}N_5O_2$: 369.1226; found: 369.1216.

3-(4-Methoxyphenyl)-1-phenyl-7,8-dihydro-3H-pyrazolo[4′,3′:5,6]pyrido[3,4-d]pyridazine-6,9-dione (**6g**), Deep yellow solid; yield: 81%; mp 311–313 °C; ^{1}H NMR (DMSO-*d*$_6$, 500 MHz) δ 3.85 (s, 3H, OC*H*$_3$), 7.17 (d, *J* = 11.2 Hz, 2H, Ar*H*), 7.43 (s, 3H, Ar*H*), 7.58–7.60 (m, 2H, Ar*H*), 8.03 (d, *J* = 11.2 Hz, 2H, Ar*H*), 9.38 (s, 1H, Ar*H*); ^{13}C{^{1}H} NMR (DMSO-*d*$_6$, 125 MHz) δ 55.49, 106.64, 114.38 (2 × CH), 115.59, 124.31 (2 × CH), 124.60, 126.71 (2 × CH), 127.77, 130.23 (2 × CH), 131.18, 135.03, 147.01, 149.17, 151.12, 152.63, 156.80, 158.33; FT-IR (KBr) *v*: 3435, 3226, 3065, 2886, 1650, 1590, 1535, 1516, 1441, 1362, 1252, 1170, 905 cm^{-1}; MS (EI) *m*/*z* (relative intensity): 386 (24), 385 (M$^+$, 100), 370 (13), 77 (18); HRMS (EI) m/z: [M]$^+$ Calcd for $C_{21}H_{15}N_5O_3$: 385.1175; found: 385.1180.

3-(4-Cyanophenyl)-1-phenyl-7,8-dihydro-3H-pyrazolo[4′,3′:5,6]pyrido[3,4-d]pyridazine-6,9-dione (**6h**), Yellow solid; yield: 73%; mp 344–347 °C; ^{1}H NMR (DMSO-*d*$_6$, 500 MHz) δ 7.43–7.44 (m, 3H, Ar*H*), 7.60–7.61 (m, 2H, Ar*H*), 8.08 (d, *J* = 6.6 Hz, 2H, Ar*H*), 8.56 (d, *J* = 6.6 Hz, 2H, Ar*H*), 9.45 (s, 1H, Ar*H*); ^{13}C{^{1}H} NMR (DMSO-*d*$_6$, 125 MHz) δ 108.84, 118.51, 121.39, 121.70 (2 × CH), 126.74 (2 × CH), 128.07, 128.63, 130.08 (2 × CH), 130.15, 133.62 (2 × CH), 134.51, 141.67, 148.75, 149.75, 151.87, 155.15, 156.71; FT-IR (KBr) *v*: 3397, 3284, 3056, 2228, 1606, 1569, 1516, 1430, 1400, 1317, 905 cm^{-1}; MS (EI) *m*/*z* (relative intensity): 381 (26), 380 (M$^+$, 100), 379 (23), 295 (17), 102 (13), 77(29); HRMS (EI) m/z: [M]$^+$ Calcd for $C_{21}H_{12}N_6O_2$: 380.1022; found: 380.1030.

3-(4-Nitrophenyl)-1-phenyl-7,8-dihydro-3H-pyrazolo[4′,3′:5,6]pyrido[3,4-d]pyridazine-6,9-dione (**6i**), Yellow solid; yield: 69%; mp 340–342 °C; ^{1}H NMR (DMSO-*d*$_6$, 500 MHz) δ 7.41–7.44 (m, 3H, Ar*H*), 7.61 (d, *J* = 6.3 Hz, 2H, Ar*H*), 8.48 (d, *J* = 8.3 Hz, 2H, Ar*H*), 8.67 (d, *J* = 8.3 Hz, 2H, Ar*H*), 9.47 (s, 1H, Ar*H*), 12.11 (br, 1H, N*H*); ^{13}C{^{1}H} NMR (DMSO-*d*$_6$, 125 MHz) δ 113.65, 121.61 (2 × CH), 124.52, 125.13 (2 × CH), 126.85 (2 × CH), 128.22, 130.12 (2 × CH), 130.27, 134.47, 143.27, 145.04, 149.17, 149.92, 152.09, 154.07, 158.18; FT-IR (KBr) *v*: 3435, 1637, 1596, 1522, 1341, 1112, 905 cm^{-1}; MS (EI) *m*/*z*(relative intensity): 401 (23), 400 (M$^+$, 100), 370 (22), 315 (11), 77(19); HRMS (EI) m/z: [M]$^+$ Calcd for For $C_{20}H_{12}N_6O_4$: 400.0920; found: 400.0919.

3-(3-Chlorophenyl)-7,8-dihydro-3H-pyrazolo[4′,3′:5,6]pyrido[3,4-d]pyridazine-6,9-dione (**6j**), Light yellow solid; yield: 71%; mp 351–352 °C; ^{1}H NMR (DMSO-d_6, 500 MHz) δ 7.51 (d, *J* = 8.03 Hz, 1H, Ar*H*), 7.66 (t, *J* = 8.0 Hz, 1H, Ar*H*), 8.28 (d, *J* = 8.0 Hz, 1H, Ar*H*), 8.39 (s, 1H, Ar*H*), 8.88 (s, 1H, Ar*H*), 9.40 (s, 1H, Ar*H*), 10.20 (br, 1H, N*H*); ^{13}C{^{1}H} NMR (DMSO-d_6, 125 MHz) δ 109.53, 119.80, 120.84, 126.68 (2 × C), 131.14 (CH + C), 133.57, 136.04, 139.62, 149.36, 150.78, 152.47, 155.87; FT-IR (KBr) *v*: 3433, 3294, 3168, 2974, 1639, 1594, 1568, 1487, 1448, 1274, 1218, 1125 cm^{-1}; MS (EI) *m/z* (relative intensity): 315 (M^+ + 2, 35), 314 (28), 313 (M^+, 100), 278 (12), 255 (13), 227 (21), 111(12), 75 (11); HRMS (EI) m/z: $[M]^+$ Calcd for $C_{14}H_8ClN_5O_2$: 313.0367; found: 313.0367.

1.3. Standard procedure for synthesis of N-aminopyrazolopyrrolopyridine diones (7a–i)

The reliable procedure were involved the treatment of 1,3-diarylpyrazolopyrrolopyridine-6,8-diones (**11a–i**, 1.0 equiv.) with hydrazine monohydrate (~5.0 equiv.) in EtOH/H_2O (2.0 mL/2.0 mL) in ice-bath to room temperature within 48 h. When the reaction was completed, the reaction mixture was added to water (10 mL) for precipitation. The precipitate was filtered, washed with cold water (10 mL) and *n*-hexane/EA (1/2, 15 mL) to give the corresponding crude *N*-aminophthalimides **7a–i**. The crude desired products **7a–i** were recrystallized in acetone/THF (1/4) solution to obtain the pure *N*-aminophthalimides **7a–i** in 71– 87 % yields.

7-Amino-1,3-diphenylpyrazolo[3,4-b]pyrrolo[3,4-d]pyridine-6,8-(3H,7H)-dione (**7a**), White solid; yield: 83%; mp 217–219 °C; ^{1}H NMR (DMSO-d_6, 400 MHz) δ 4.55 (br, 2H, NH_2), 7.42 (t, *J* = 7.5 Hz, 1H, Ar*H*), 7.49–7.55 (m, 3H, Ar*H*), 7.60–7.65 (m, 4H, Ar*H*), 8.25 (d, *J* = 8.1 Hz, 2H, Ar*H*), 8.82 (s, 1 H, Ar*H*); ^{13}C{^{1}H} NMR (DMSO-d_6, 100 MHz) δ 111.65, 121.43 (2 × CH), 123.37, 126.80, 128.27 (2 × CH), 128.58 (2 × CH), 128.96, 129.41 (2 × CH), 132.04, 138.44, 139.23, 146.02, 148.82, 150.61, 164.18, 164.49; FT-IR (KBr) *v*: 3275, 3208, 3172, 3035, 1633.0, 1572, 1518.7, 1501 cm^{-1}; MS (EI) *m/z* (relative intensity): 356 (20), 355 (M^+, 100), 354 (18), 270 (13), 77.0(18); HRMS (EI) m/z: $[M]^+$ Calcd for $C_{20}H_{13}N_5O_2$: 355.1069; found: 355.1060.

7-Amino-3-(2-chlorophenyl)-1-phenylpyrazolo[3,4-b]pyrrolo[3,4-d]pyridine-6,8-(3H,7H)-dione (**7b**), Yellow solid; yield: 71%; mp 165–166 °C; ^{1}H NMR (DMSO-d_6, 500 MHz) δ 4.52 (br, 2H, NH_2), 7.48 (d, *J* = 7.0 Hz, 1H, Ar*H*), 7.52 (d, *J* = 7.1 Hz, 2H, Ar*H*), 7.60–7.68 (m, 4H, Ar*H*), 7.74 (d, *J* = 7.7 Hz, 1H, Ar*H*), 7.79 (d, *J* =7.7 Hz, 1H, Ar*H*), 8.71 (s, 1H, Ar*H*); ^{13}C{^{1}H} NMR (DMSO-d_6, 125 MHz) δ 108.50, 122.16, 122.88, 123.33, 127.72 (2 × CH), 129.27 (3 × CH), 129.72 (2 × CH), 131.26, 131.46, 136.60, 136.98, 143.86, 145.72, 153.20, 166.81, 168.47; FT-IR (KBr) *v*: 3337, 3296, 2952, 2920, 1778, 1740, 1498, 1375, 1315, 1014 cm^{-1}; MS (EI) *m/z* (relative intensity): 391 (M^+ + 2, 32), 390 (22), 389 (M^+, 100), 355 (18), 354 (90), 304 (12), 268 (11), 77 (18); HRMS (EI) m/z: $[M]^+$ Calcd for $C_{20}H_{12}ClN_5O_2$: 389.0680; found: 389.0672.

7-Amino-3-(3-chlorophenyl)-1-phenylpyrazolo[3,4-b]pyrrolo[3,4-d]pyridine-6,8-(3H,7H)-dione (**7c**), Yellow solid; yield: 73%; mp 173–175 °C; ^{1}H NMR (DMSO-d_6, 400 MHz) δ 4.55 (br, 2H, NH_2), 7.47–7.54 (m, 4H, ArH), 7.64–7.67 (m, 3H, ArH), 8.31 (d, *J* = 8.9 Hz, 1H, Ar*H*), 8.42 (s, 1H, Ar*H*), 8.86 (s, 1H, Ar*H*); ^{13}C{^{1}H} NMR (DMSO-d_6, 100 MHz) δ 112.05, 119.33, 120.34, 123.72, 126.32, 128.28 (2 × CH), 128.57 (2 × CH), 129.11, 131.21, 131.73, 133.65, 139.41, 139.64, 146.61, 148.98, 150.72, 163.96, 164.33; FT-IR (KBr) *v*: 3264, 3168, 3034, 1649, 1614, 1595, 1488, 1431, 1300, 803 cm^{-1}; MS (EI) *m/z* (relative intensity): 391 (M^+ + 2, 33), 390 (24), 389 (M^+, 100), 374 (13), 304 (11), 77 (14); HRMS (EI) m/z: $[M]^+$ Calcd for $C_{20}H_{12}ClN_5O_2$: 389.0680; found: 389.0688.

7-Amino-3-(4-chlorophenyl)-1-phenylpyrazolo[3,4-b]pyrrolo[3,4-d]pyridine-6,8-(3H,7H)-dione (**7d**), Light yellow solid; yield: 81%; mp 226–227 °C; ^{1}H NMR (DMSO-d_6, 500 MHz) δ 4.55 (br, 2H, NH_2), 7.48–7.55 (m, 3H, Ar*H*), 7.64 (d, *J* = 7.6 Hz, 2H, Ar*H*), 7.69 (d, *J* = 8.4 Hz, 2H, Ar*H*), 8.35 (d, *J* = 8.4 Hz, 2H, Ar*H*), 8.84 (s, 1 H, Ar*H*); ^{13}C{^{1}H} NMR (DMSO-d_6, 125 MHz) δ 111.85, 122.54 (2 × CH), 123.55, 128.24 (2 × CH), 128.53 (2 × CH), 129.03, 129.36 (2 × CH), 130.67, 131.81, 137.31, 139.34, 146.35, 148.88, 150.57, 163.99, 164.34; FT-IR (KBr) *v*: 3275, 3207, 3170, 1633, 1499, 828 cm^{-1}; MS (EI) *m/z* (relative intensity): 391 (M^++ 2, 34), 390 (28), 389 (M^+, 100), 388 (15), 304 (12), 77 (13); HRMS (EI) m/z: $[M]^+$ Calcd for $C_{20}H_{12}ClN_5O_2$: 389.0680; found: 389.0686.

7-Amino-3-(4-bromophenyl)-1-phenylpyrazolo[3,4-b]pyrrolo[3,4-d]pyridine-6,8-(3H,7H)-dione (**7e**), Yellow solid; yield: 79%; mp 235–239 °C; ^{1}H NMR (DMSO-d_6, 500 MHz) δ 4.55 (br, 2H, NH_2), 7.49–7.55 (m, 3H, Ar*H*), 7.64 (d, *J* = 6.9 Hz, 2H, Ar*H*), 7.82 (d, *J* = 8.7 Hz, 2H, Ar*H*), 8.29 (d, *J* = 8.7 Hz, 2H,

Ar*H*), 8.84 (s, 1 H, Ar*H*); $^{13}C\{^1H\}$ NMR (DMSO-*d*$_6$, 125 MHz) δ 111.91, 118.95, 122.81 (2 × CH), 123.55, 128.25 (2 × CH), 128.54 (2 × CH), 129.04, 131.80, 132.28 (2 × CH), 137.75, 139.33, 146.40, 148.90, 150.59, 164.00, 164.35; FT-IR (KBr) *v*: 3275, 3208, 3170, 3071, 3037, 1632, 1495, 826 cm^{-1}; MS (EI) *m/z* (relative intensity): 436 (22), 435 (M^+ + 2, 100), 354 (33), 433 (M^+, 99), 432 (12), 420 (19), 419 (11), 418 (19), 354 (11), 350 (10), 348 (11), 268 (17), 77 (26); HRMS (EI) m/z: $[M]^+$ Calcd for $C_{20}H_{12}BrN_5O_2$: 433.0174; found: 433.0171.

7-Amino-1-phenyl-3-(p-tolyl)pyrazolo[3,4-b]pyrrolo[3,4-d]pyridine-6,8-(3H,7H)-dione (**7f**), Yellow solid; yield: 86%; mp 232–233 °C; ^1H NMR (DMSO-*d*$_6$, 500 MHz) δ 2.40 (s, 3H, C*H*$_3$), 4.55 (br, 2H, N*H*$_2$), 7.42 (d, *J* = 8.0 Hz, 2H, Ar*H*), 7.49 (d, *J* = 7.3 Hz, 1H, Ar*H*), 7.52 (t, *J* = 7.3 Hz, 2H, Ar*H*), 7.64 (d, *J* = 7.3 Hz, 2H, Ar*H*), 8.12 (d, *J* = 8.0 Hz, 2H Ar*H*), 8.81 (s, 1H, Ar*H*); $^{13}C\{^1H\}$ NMR (DMSO-*d*$_6$, 125 MHz) δ 20.63, 111.44, 121.35(2 × CH), 123.18, 128.21 (2 × CH), 128.54 (2 × CH), 128.85, 129.73 (2 × CH), 132.09, 136.06, 136.18, 139.13, 145.69, 148.72, 150.44, 164.20, 164.50; FT-IR (KBr) *v*: 3276, 3209, 3171, 3032, 1634, 1517 cm^{-1}; MS (EI) *m/z* (relative intensity): 370 (25), 369 (M^+, 100), 368 (13), 354 (21), 284 (12), 207 (10), 91.1(11), 77.1(13); HRMS (EI) m/z: $[M]^+$ Calcd for $C_{21}H_{15}N_5O_2$: 369.1226; found: 369.1231.

7-Amino-3-(4-methoxyphenyl)-1-phenylpyrazolo[3,4-b]pyrrolo[3,4-d]pyridine-6,8-(3H,7H)-dione (**7g**), Yellow solid; yield: 87%; mp 331–332 °C; ^1H NMR (DMSO-*d*$_6$, 500 MHz) δ 3.83 (s, 3H, OC*H*$_3$), 4.53 (br, 2H, N*H*$_2$), 7.17 (d, *J* = 8.7 Hz, 2H, Ar*H*), 7.48–7.53 (m, 3H, Ar*H*), 7.62 (d, *J* = 7.1 Hz, 2H, Ar*H*), 8.06 (d, *J* = 8.7 Hz, 2H, Ar*H*), 8.78 (s, 1H, ArH); $^{13}C\{^1H\}$ NMR (DMSO-*d*$_6$, 125 MHz) δ 55.64, 111.24, 114.62 (2 × CH), 123.14, 123.51 (2 × CH), 128.37 (2 × CH), 128.65 (2 × CH), 128.97, 131.60, 132.25, 139.14, 145.54, 148.83, 150.44, 158.16, 164.43, 164.73; FT-IR (KBr) *v*: 3215, 3066, 3004, 2963, 2935, 2837, 1639, 1577, 1516, 1462, 1443, 1252 cm^{-1}; MS (EI) *m/z* (relative intensity): 386 (21), 385 (M^+, 100), 370 (14), 77.0(10); HRMS (EI) m/z: $[M]^+$ Calcd for $C_{21}H_{15}N_5O_3$: 385.1175; found: 385.1182.

7-Amino-3-(4-cyanophenyl)-1-phenylpyrazolo[3,4-b]pyrrolo[3,4-d]pyridine-6,8-(3H,7H)-dione (**7h**), Yellow solid; yield: 73%; mp 333–335 °C; ^1H NMR (DMSO-*d*$_6$, 500 MHz) δ 4.55 (br, 2H, N*H*$_2$), 7.50–7.55 (m, 3H, Ar*H*), 7.65 (d, *J* = 7.1 Hz, 2H, Ar*H*), 8.09 (d, *J* = 8.5 Hz, 2H, Ar*H*), 8.61 (d, *J* = 8.5 Hz, 2H, Ar*H*), 8.87 (s, 1 H, Ar*H*); $^{13}C\{^1H\}$ NMR (DMSO-*d*$_6$, 125 MHz) δ 108.41, 112.58, 118.68, 120.75 (2 × CH), 124.12, 128.35 (2 × CH), 128.59 (2 × CH), 129.33, 131.55, 133.84 (2 × CH), 139.53, 141.99, 147.47, 149.11, 151.12, 163.83, 164.27; FT-IR (KBr) *v*: 3330, 3274, 2227, 1665, 1635, 1607, 1518, 1409, 1255, 844 cm^{-1}; MS (EI) *m/z* (relative intensity): 381 (22), 380 (M^+, 100), 379 (18), 295 (13), 77(14); HRMS (EI) m/z: $[M]^+$ Calcd for $C_{21}H_{12}N_6O_2$: 380.1022; found: 380.1023.

7-Amino-3-(4-nitrophenyl)-1-phenylpyrazolo[3,4-b]pyrrolo[3,4-d]pyridine-6,8-(3H,7H)-dione (**7i**), Yellow solid; yield: 74%; mp 281–282 °C; ^1H NMR (DMSO-*d*$_6$, 500 MHz) δ 7.53–7.57 (m, 3H, Ar*H*), 7.93 (d, *J* = 6.5 Hz, 2H, Ar*H*), 8.48 (d, *J* = 9.2 Hz, 2H, Ar*H*), 8.67 (d, *J* = 9.2 Hz, 2H, Ar*H*), 9.19 (s, 1H, Ar*H*); $^{13}C\{^1H\}$ NMR (DMSO-*d*$_6$, 125 MHz) δ 109.57, 121.41 (2 × CH), 123.52, 125.21 (2 × CH), 127.91 (2 × CH), 129.73, 129.90 (2 × CH), 131.21, 136.89, 143.24, 144.26, 145.12, 147.11, 153.90, 166.71, 168.43; FT-IR (KBr) *v*: 3190, 3120, 3064, 1595, 1500, 1341, 1112, 857 cm^{-1}; MS (EI) *m/z* (relative intensity): 400 (M^+, 4), 386 (26), 385 (100), 338 (13), 236 (10), 77 (13); HRMS (EI) m/z: $[M]^+$ Calcd for $C_{20}H_{12}N_6O_4$: 400.0920; found: 400.0925.

1.4. Determination of the fluorescence quantum yield

The fluorescence quantum yield Φ_x was determined through the comparative method. The quinine sulfate (Φ_{st} = 0.60, λ_{ex}= 350 nm) in H_2SO_4 0.05 M was used as the standard, and it was calculated by following equation:

$$\Phi_x / \Phi_{st} = [A_{st} / A_x]\,[n_x^2 / n_{st}^2]\,[D_x / D_{st}], \quad (1)$$

Where st: standard; x: sample; Φ: quantum yield; A: absorbance at the excitation wavelength; D: area under the fluorescence spectra on an energy scale; n: the refractive index of the solution. In the process of detection, the absorbance should be controlled and lower than 0.1.

6a

1,3-Diphenyl-7,8-dihydro-3*H*-pyrazolo[4',3':5,6]pyrido[3,4-*d*]pyridazine-6,9-dione

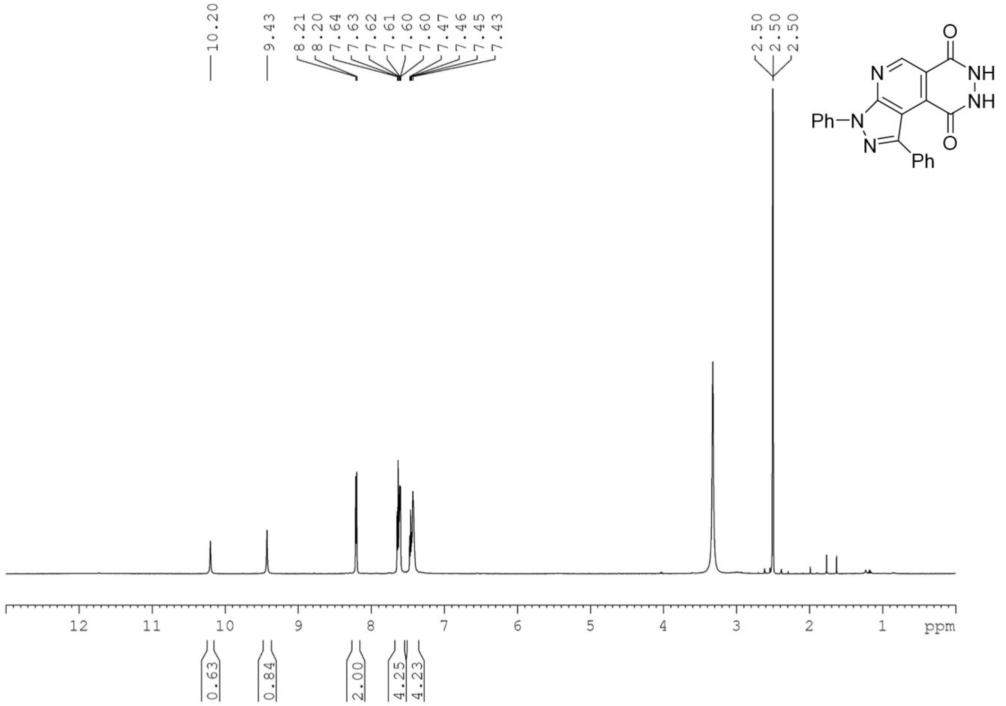

6a

1,3-Diphenyl-7,8-dihydro-3*H*-pyrazolo[4',3':5,6]pyrido[3,4-*d*]pyridazine-6,9-dione

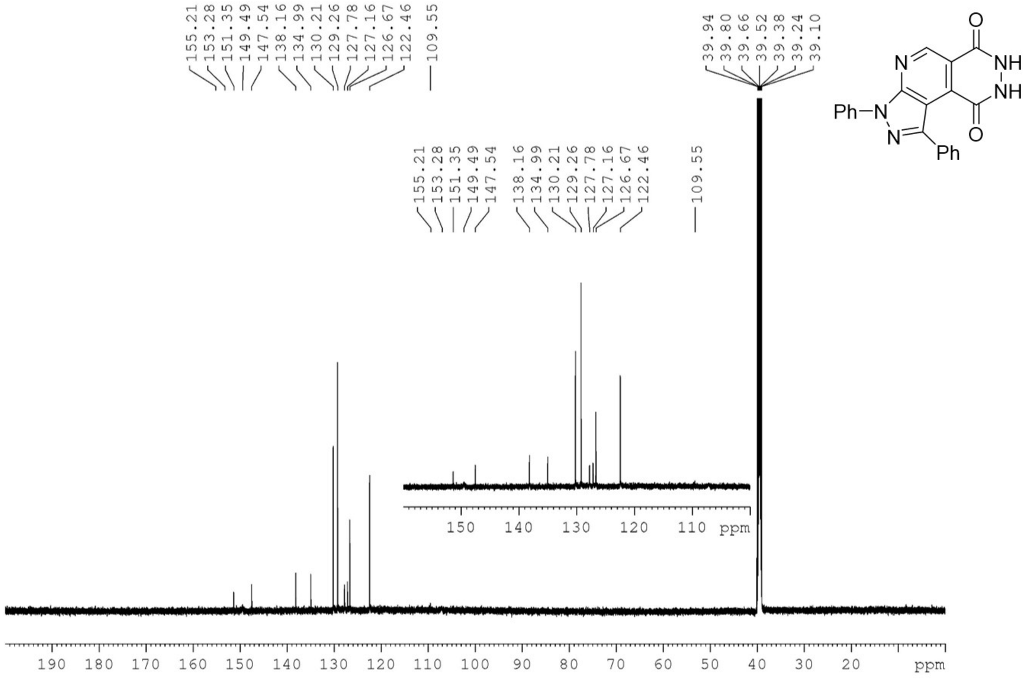

6b

3-(2-Chlorophenyl)-1-phenyl-7,8-dihydro-3*H*-pyrazolo[4',3':5,6]pyrido[3,4-*d*]pyridazine-6,9-dione

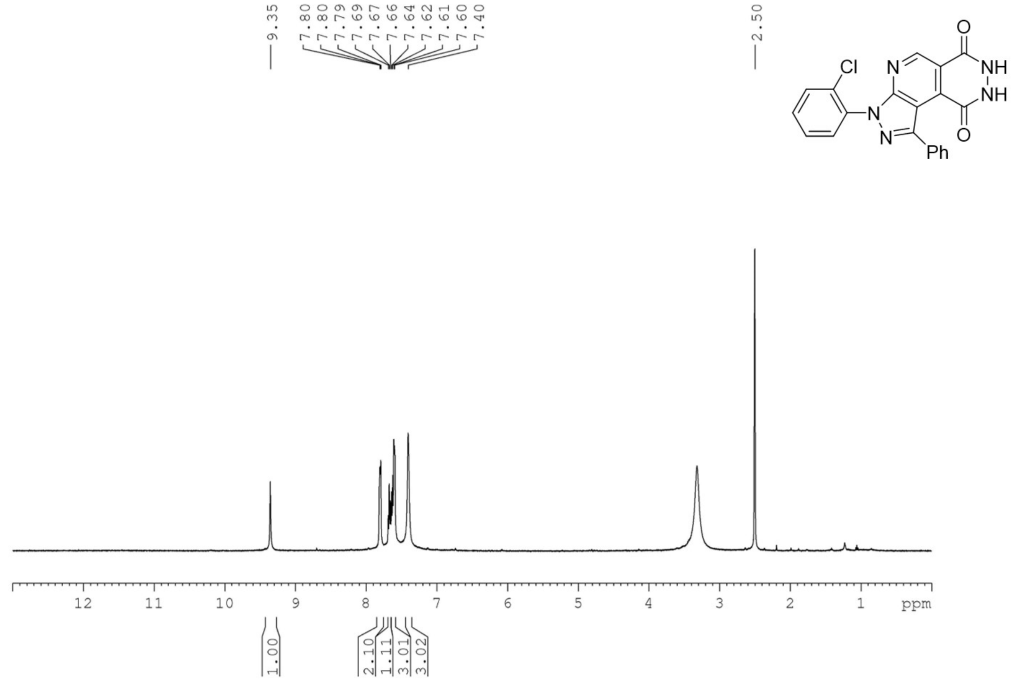

6b

3-(2-Chlorophenyl)-1-phenyl-7,8-dihydro-3*H*-pyrazolo[4',3':5,6]pyrido[3,4-*d*]pyridazine-6,9-dione

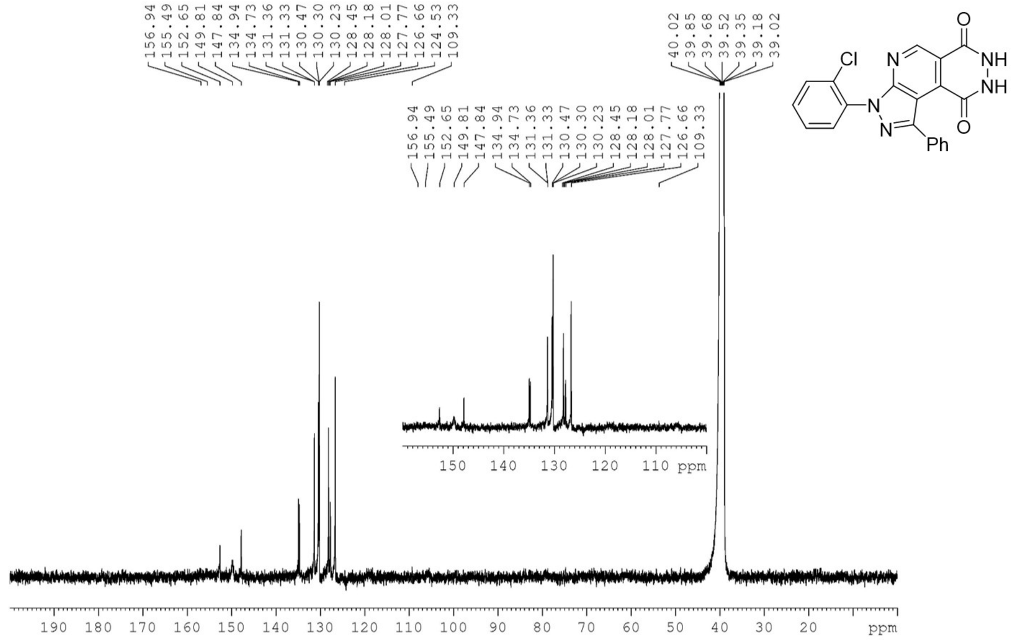

6c

3-(3-Chlorophenyl)-1-phenyl-7,8-dihydro-3*H*-pyrazolo[4',3':5,6]pyrido[3,4-*d*]pyridazine-6,9-dione

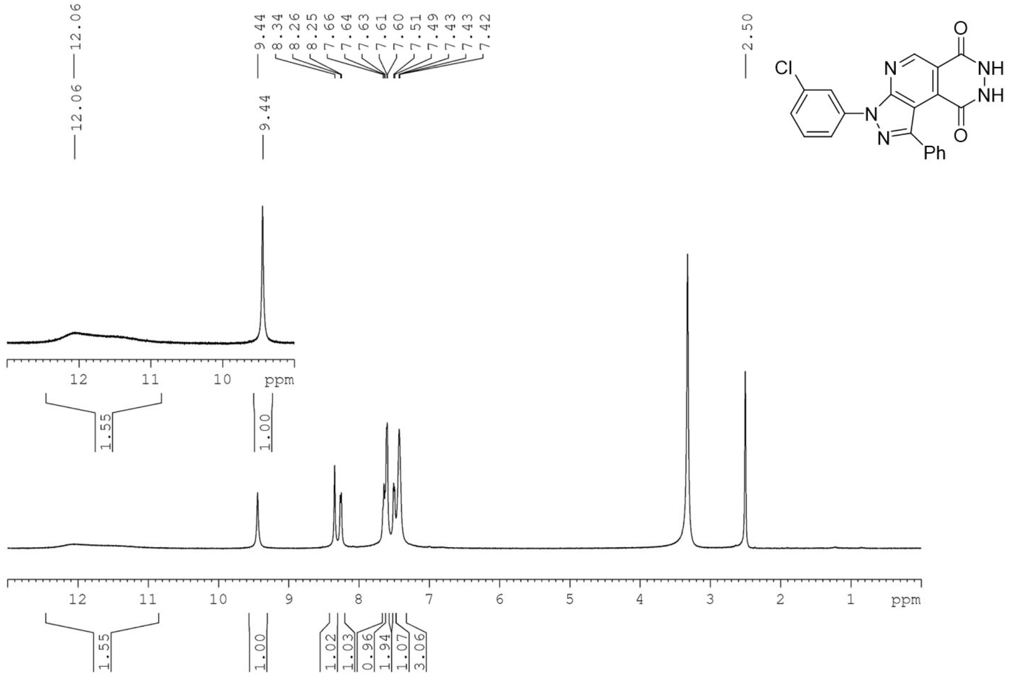

6c

3-(3-Chlorophenyl)-1-phenyl-7,8-dihydro-3*H*-pyrazolo[4',3':5,6]pyrido[3,4-*d*]pyridazine-6,9-dione

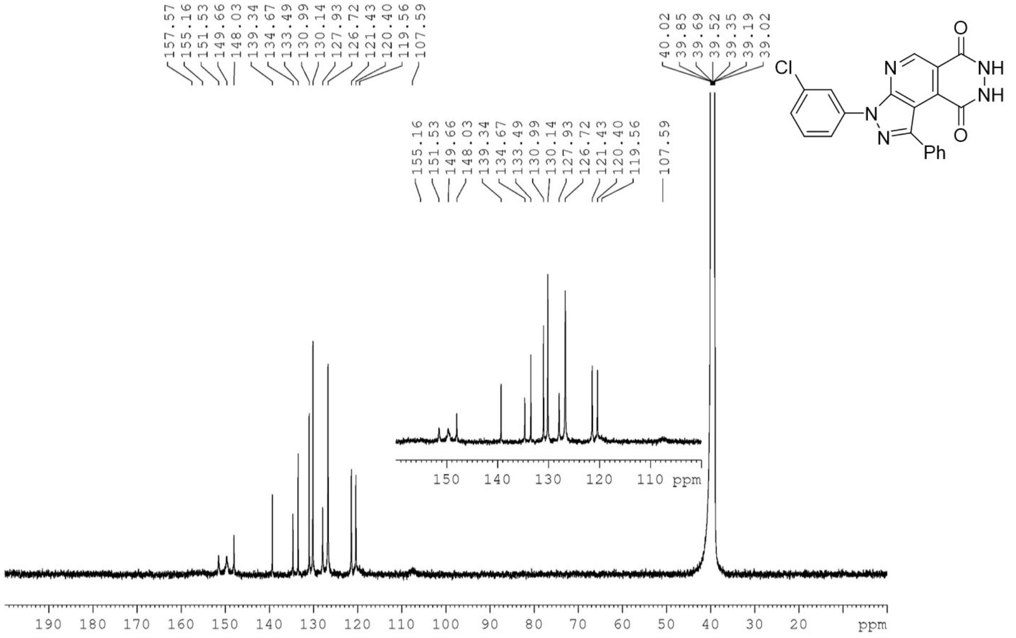

6d

3-(4-Chlorophenyl)-1-phenyl-7,8-dihydro-3*H*-pyrazolo[4',3':5,6]pyrido[3,4-*d*]pyridazine-6,9-dione

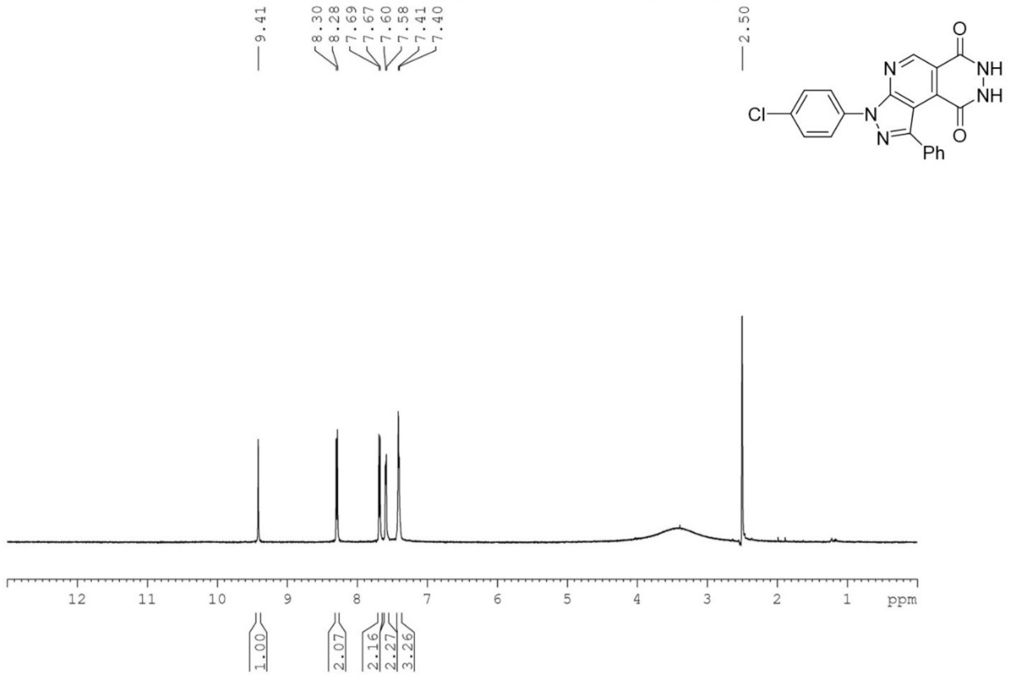

6d

3-(4-Chlorophenyl)-1-phenyl-7,8-dihydro-3*H*-pyrazolo[4',3':5,6]pyrido[3,4-*d*]pyridazine-6,9-dione

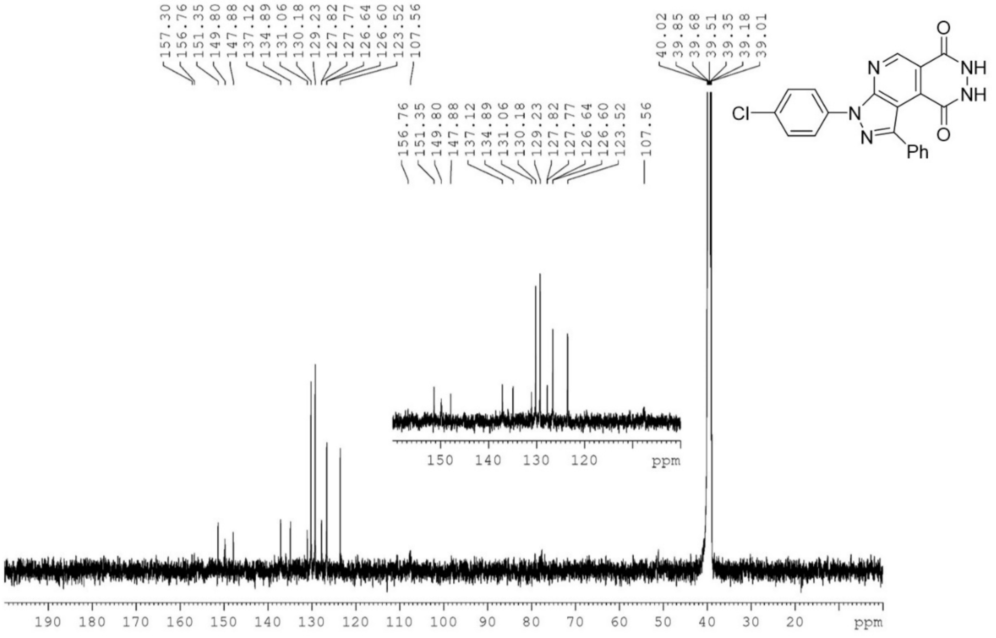

6e

3-(4-Bromophenyl)-1-phenyl-7,8-dihydro-3*H*-pyrazolo[4',3':5,6]pyrido[3,4-*d*]pyridazine-6,9-dione

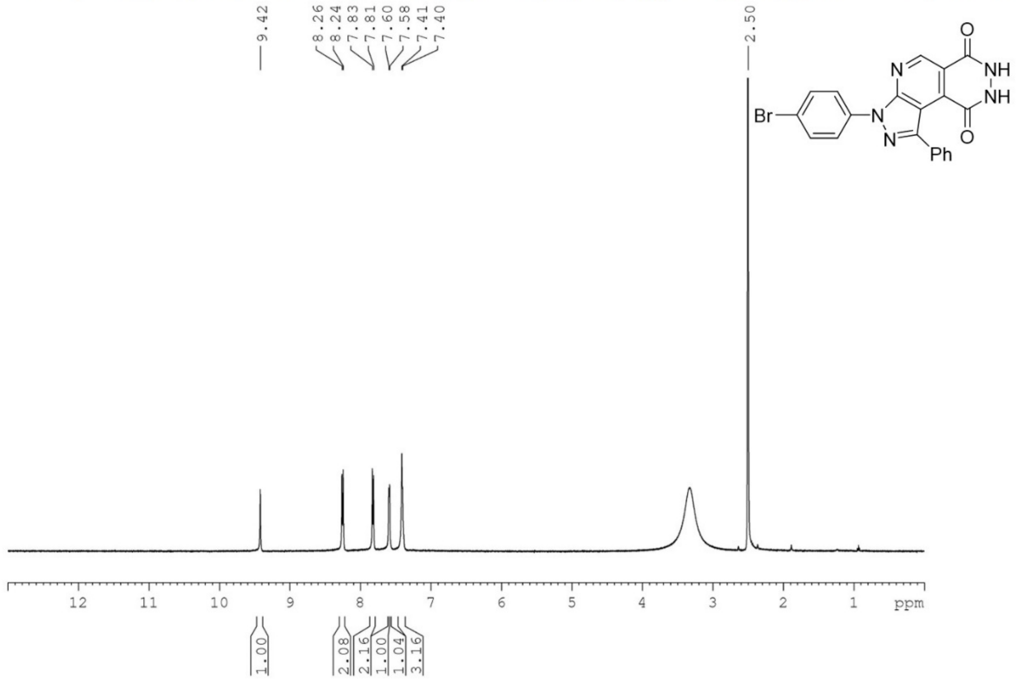

6e

3-(4-Bromophenyl)-1-phenyl-7,8-dihydro-3*H*-pyrazolo[4',3':5,6]pyrido[3,4-*d*]pyridazine-6,9-dione

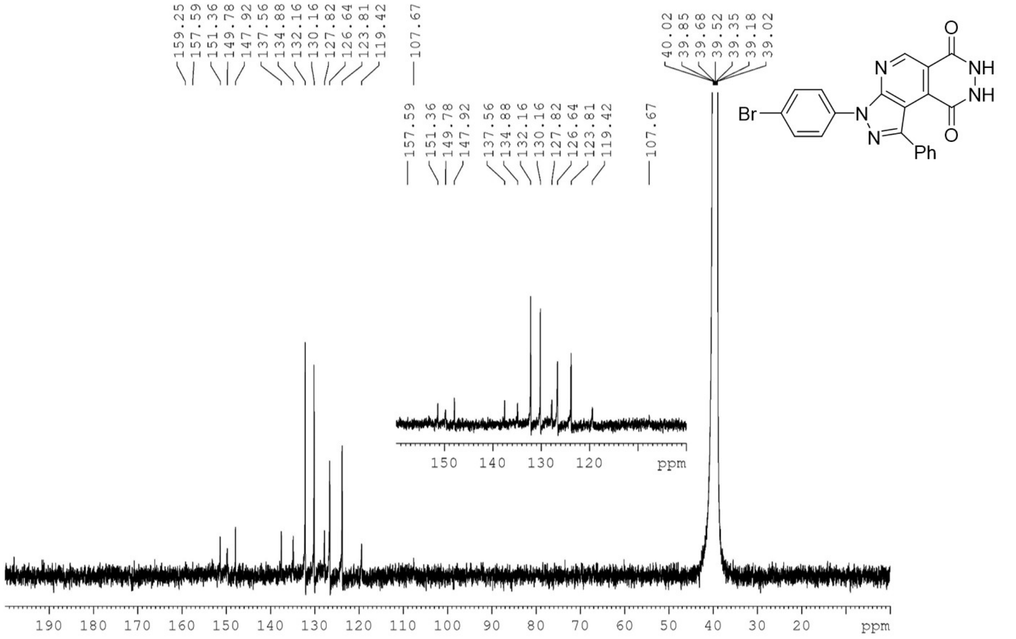

6f

1-Phenyl-3-(*p*-tolyl)-7,8-dihydro-3*H*-pyrazolo[4',3':5,6]pyrido[3,4-*d*]pyridazine-6,9-dione

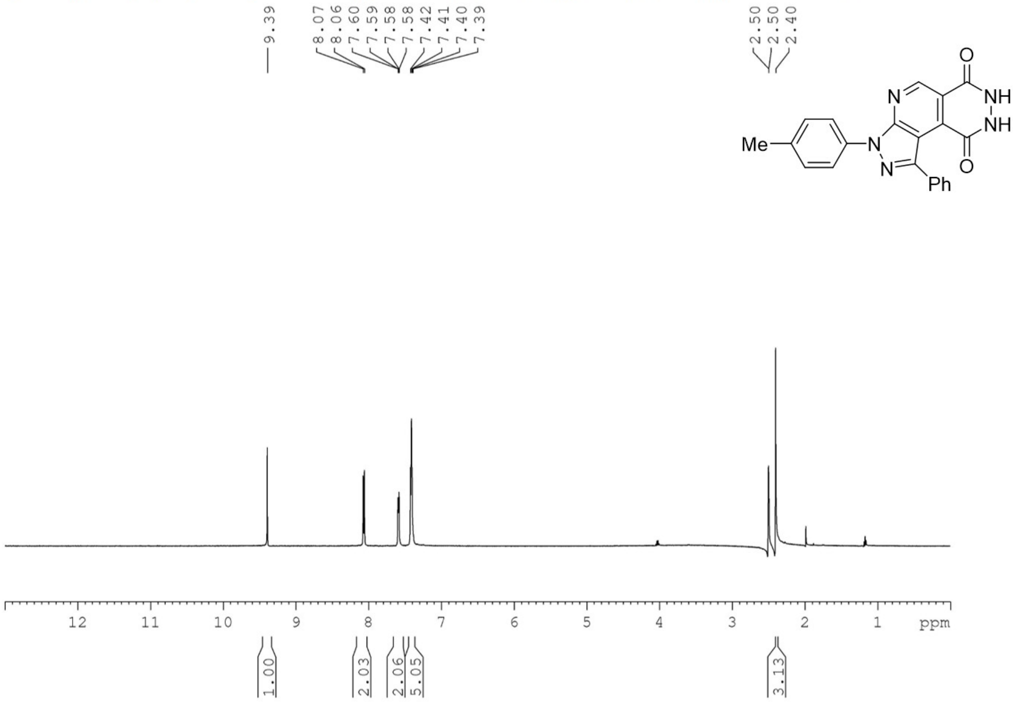

6f

1-Phenyl-3-(*p*-tolyl)-7,8-dihydro-3*H*-pyrazolo[4',3':5,6]pyrido[3,4-*d*]pyridazine-6,9-dione

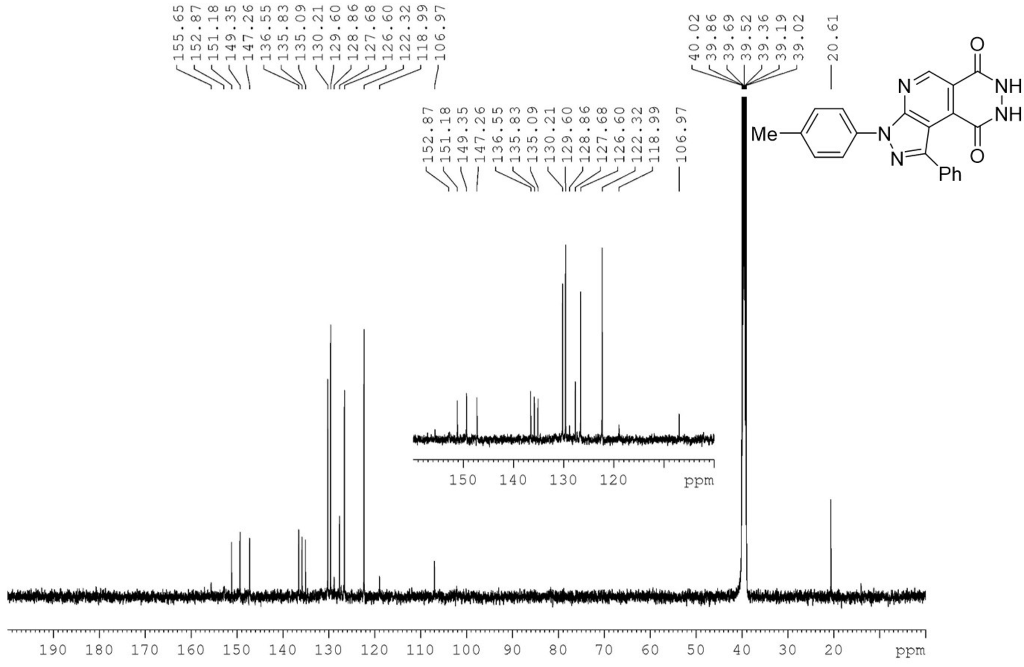

6g

3-(4-Methoxyphenyl)-1-phenyl-7,8-dihydro-3*H*-pyrazolo[4',3':5,6]pyrido[3,4-*d*] pyridazine-6,9-dione

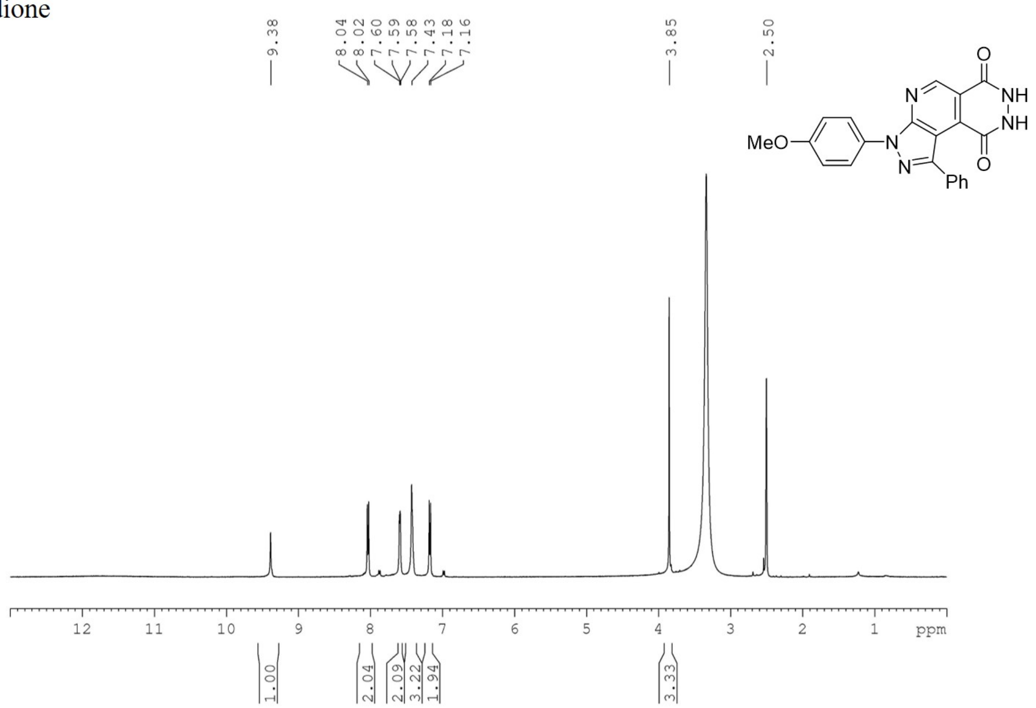

6g

3-(4-Methoxyphenyl)-1-phenyl-7,8-dihydro-3*H*-pyrazolo[4',3':5,6]pyrido[3,4-*d*] pyridazine-6,9-dione

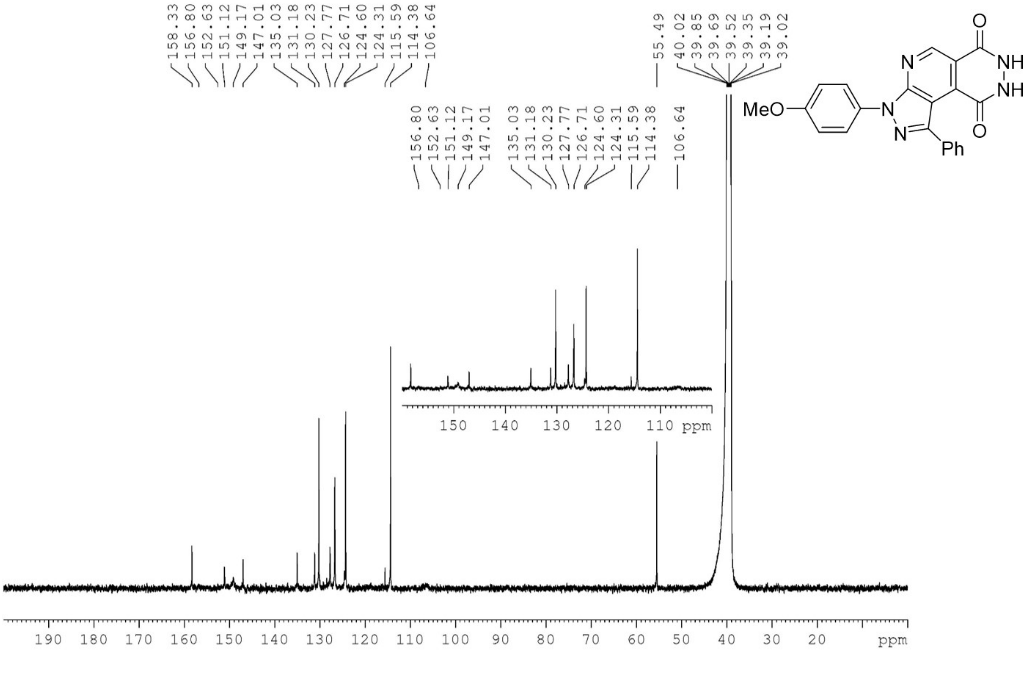

6h

3-(4-Cyanophenyl)-1-phenyl-7,8-dihydro-3*H*-pyrazolo[4',3':5,6]pyrido[3,4-*d*]pyridazine-6,9-dione

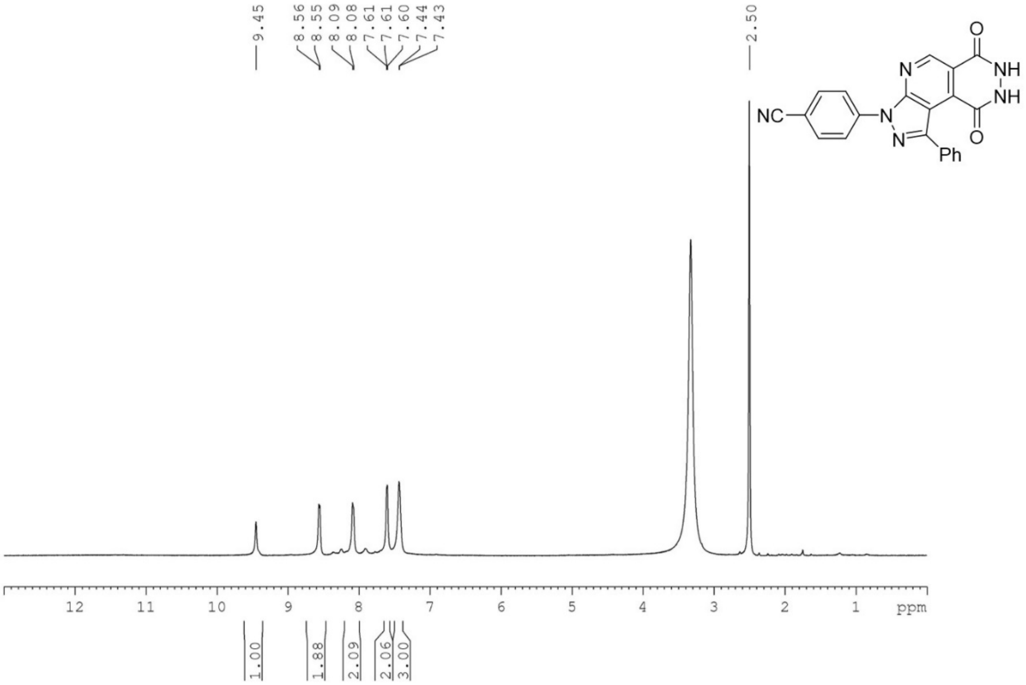

6h

3-(4-Cyanophenyl)-1-phenyl-7,8-dihydro-3*H*-pyrazolo[4',3':5,6]pyrido[3,4-*d*]pyridazine-6,9-dione

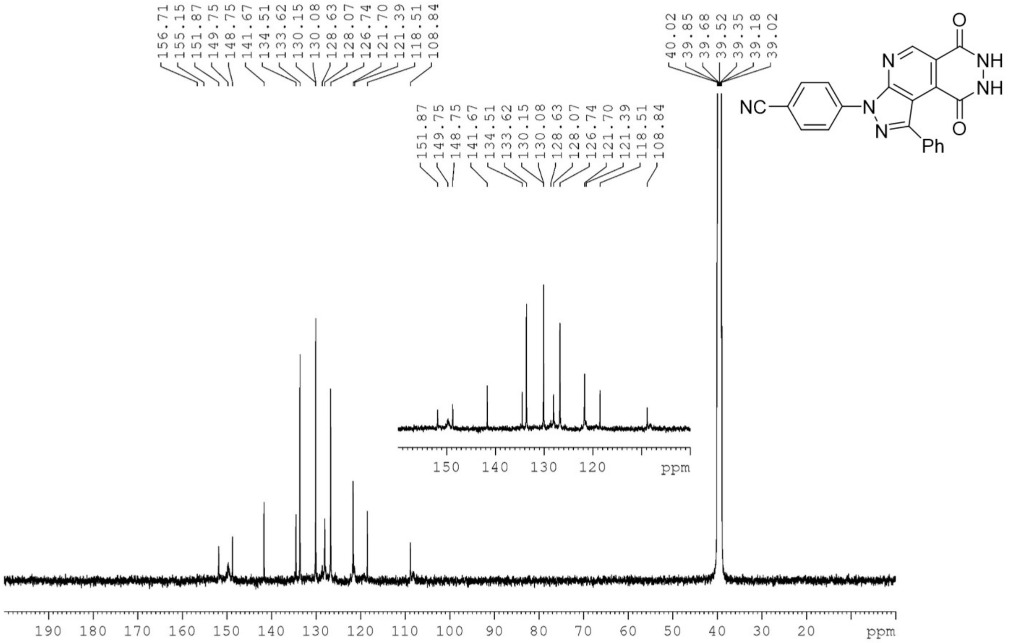

6i

3-(4-Nitrophenyl)-1-phenyl-7,8-dihydro-3*H*-pyrazolo[4',3':5,6]pyrido[3,4-*d*]pyridazine-6,9-dione

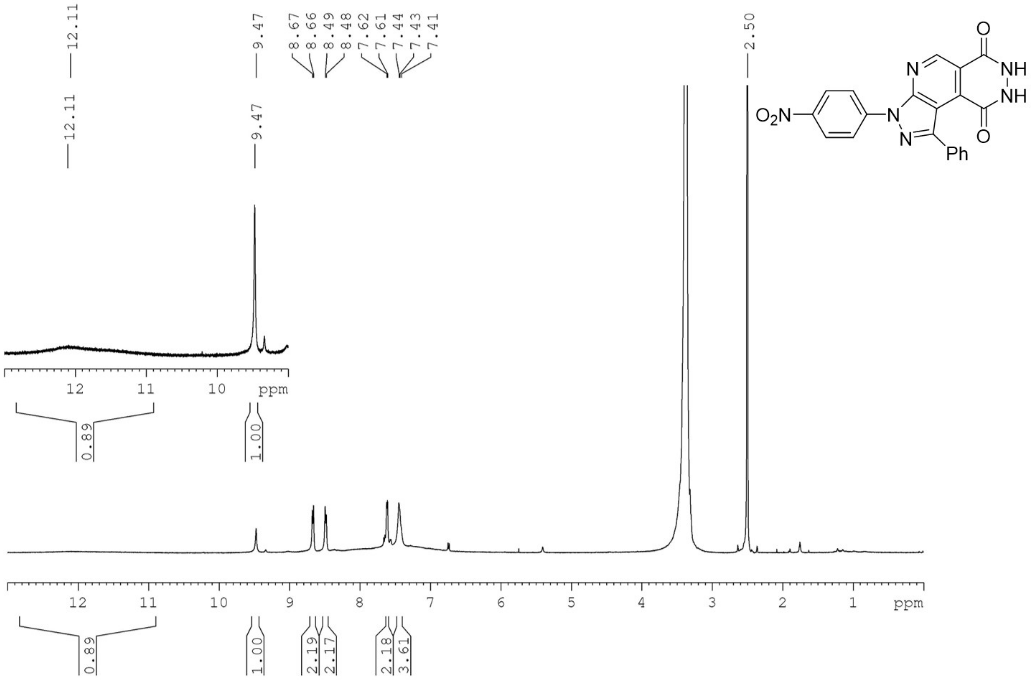

6i

3-(4-Nitrophenyl)-1-phenyl-7,8-dihydro-3*H*-pyrazolo[4',3':5,6]pyrido[3,4-*d*]pyridazine-6,9-dione

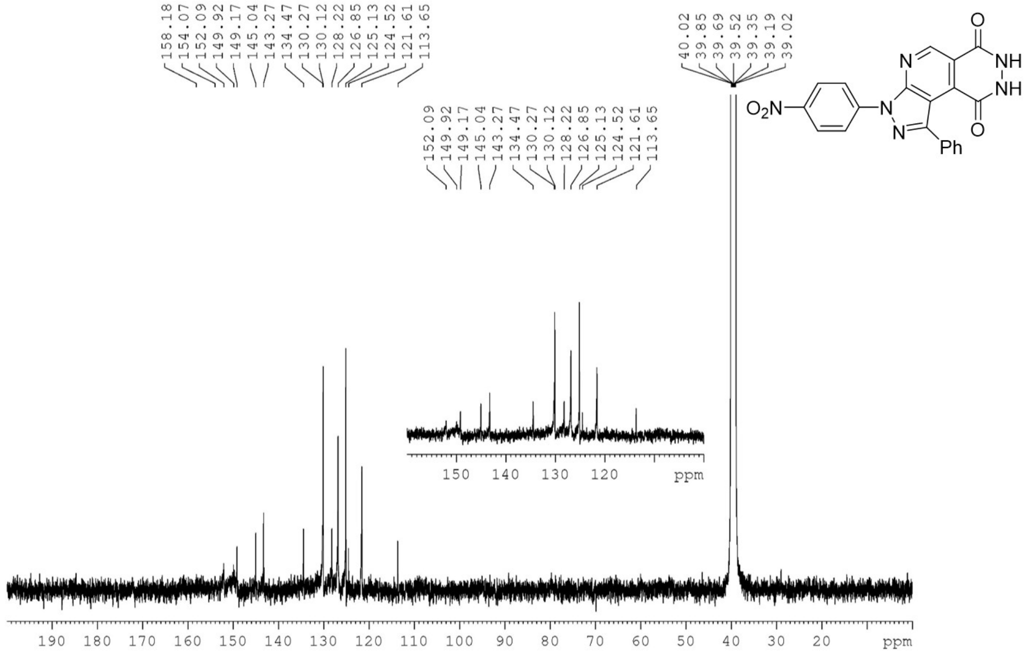

6j

3-(3-Chlorophenyl)-7,8-dihydro-3*H*-pyrazolo[4',3':5,6]pyrido[3,4-*d*]pyridazine-6,9-dione

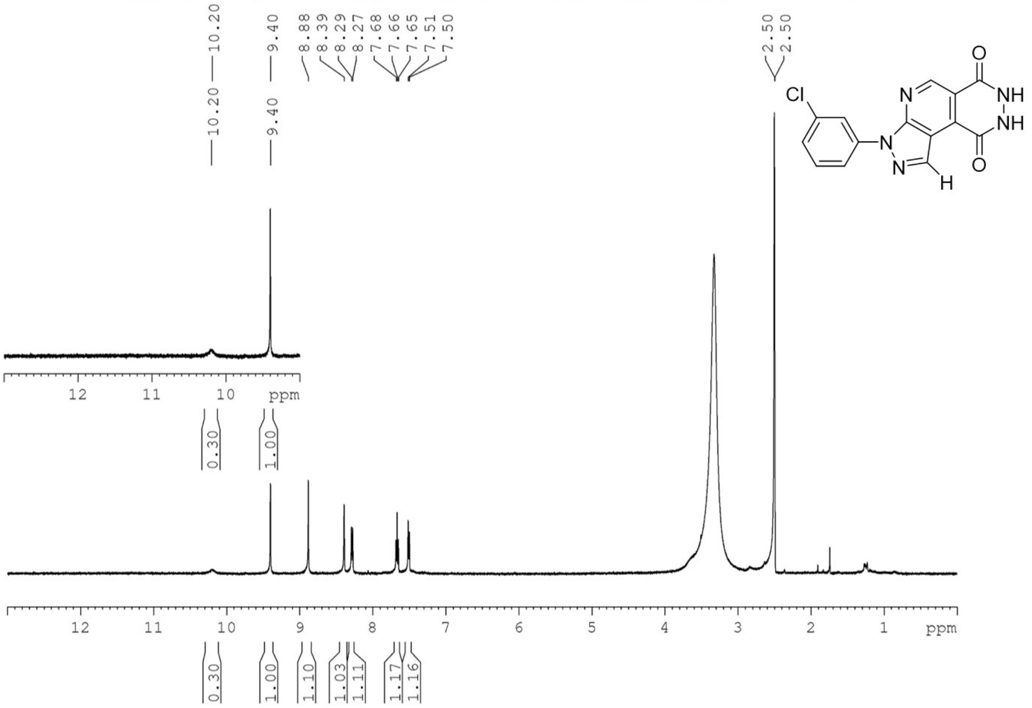

6j

3-(3-Chlorophenyl)-7,8-dihydro-3*H*-pyrazolo[4',3':5,6]pyrido[3,4-*d*]pyridazine-6,9-dione

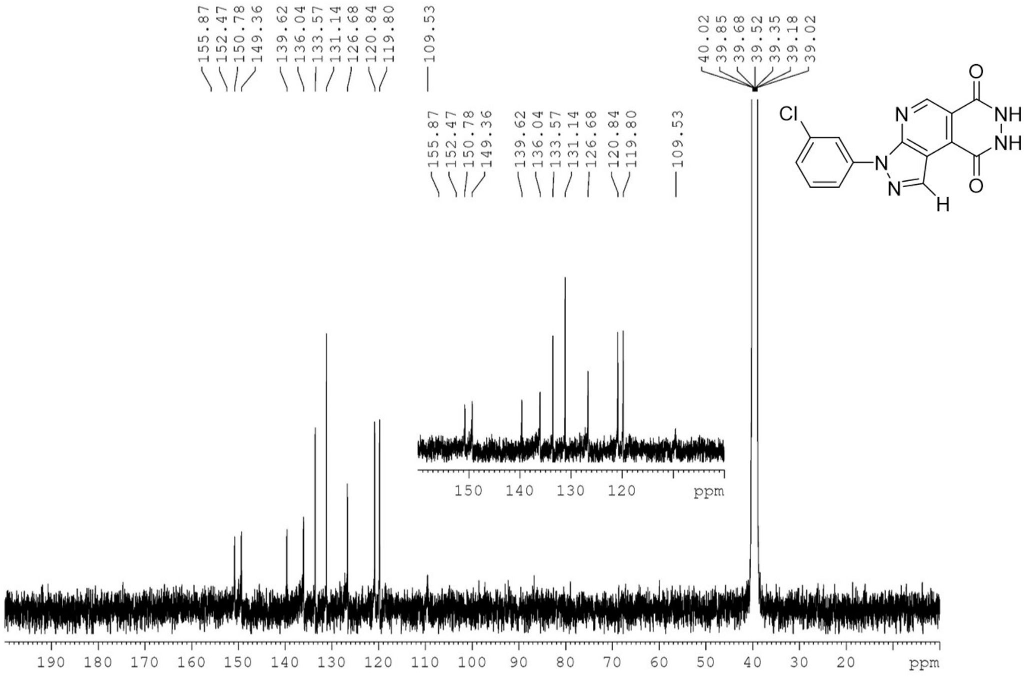

7a

7-Amino-1,3-diphenylpyrazolo[3,4-*b*]pyrrolo[3,4-*d*]pyridine-6,8(3*H*,7*H*)-dione

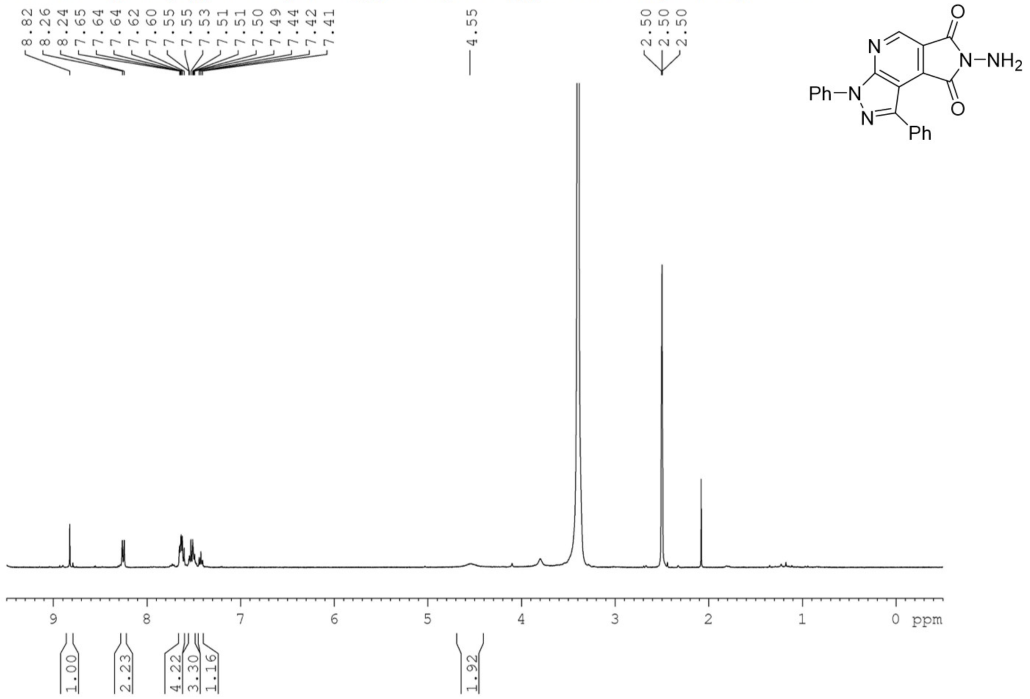

7a

7-Amino-1,3-diphenylpyrazolo[3,4-*b*]pyrrolo[3,4-*d*]pyridine-6,8(3*H*,7*H*)-dione

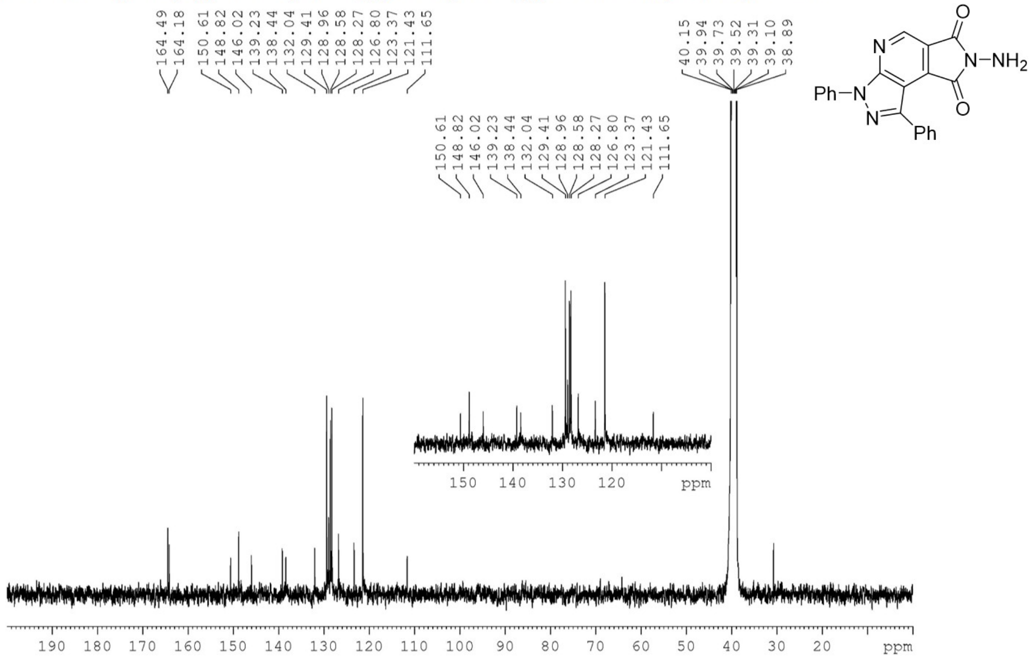

7b

7-Amino-3-(2-chlorophenyl)-1-phenylpyrazolo[3,4-*b*]pyrrolo[3,4-*d*] pyridine-6,8(3*H*,7*H*)-dione

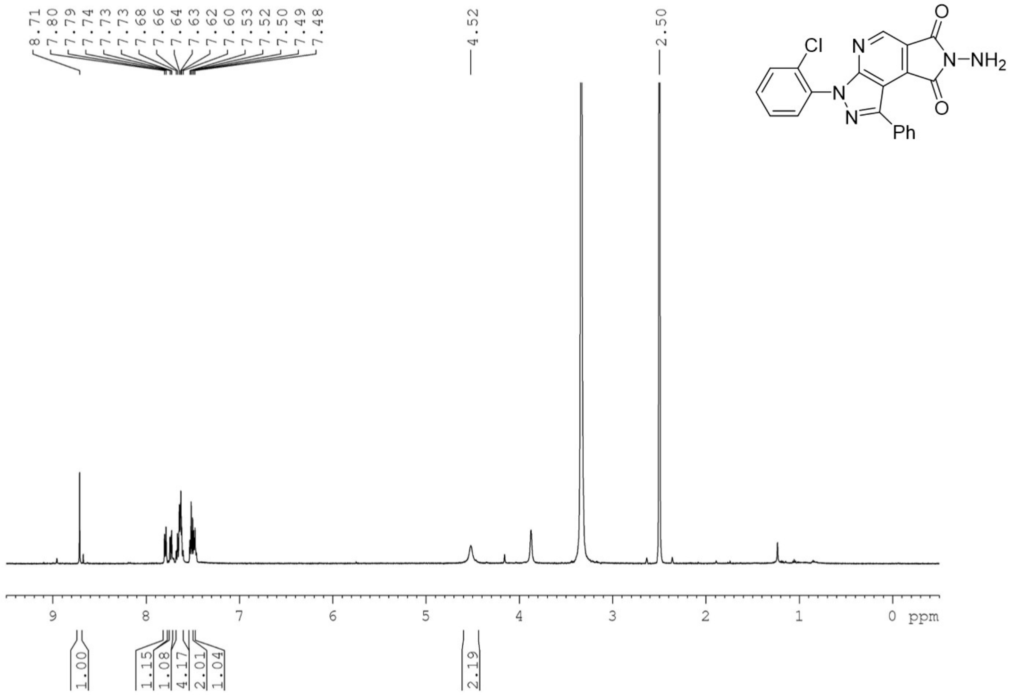

7b

7-Amino-3-(2-chlorophenyl)-1-phenylpyrazolo[3,4-*b*]pyrrolo[3,4-*d*] pyridine-6,8(3*H*,7*H*)-dione

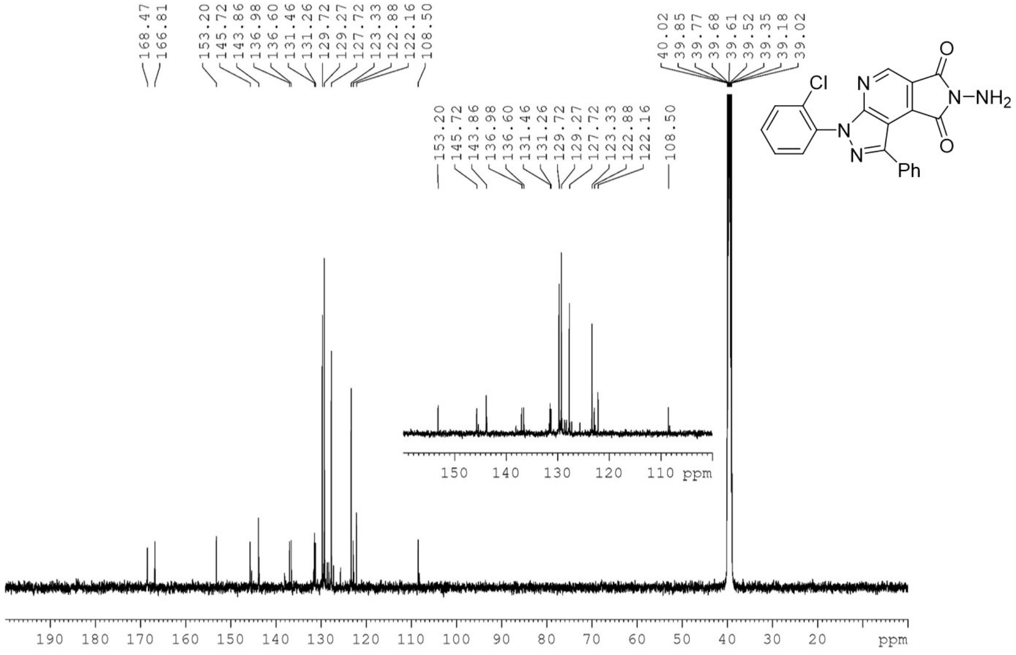

7c

7-Amino-3-(3-chlorophenyl)-1-phenylpyrazolo[3,4-*b*]pyrrolo[3,4-*d*] pyridine-6,8(3*H*,7*H*)-dione

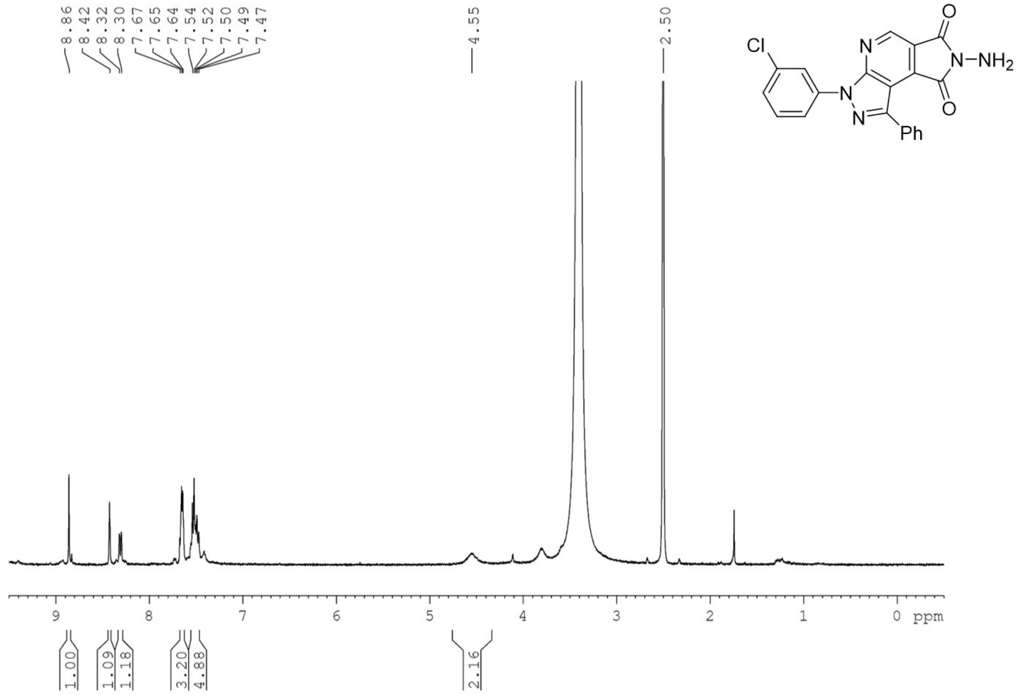

7c

7-Amino-3-(3-chlorophenyl)-1-phenylpyrazolo[3,4-*b*]pyrrolo[3,4-*d*] pyridine-6,8(3*H*,7*H*)-dione

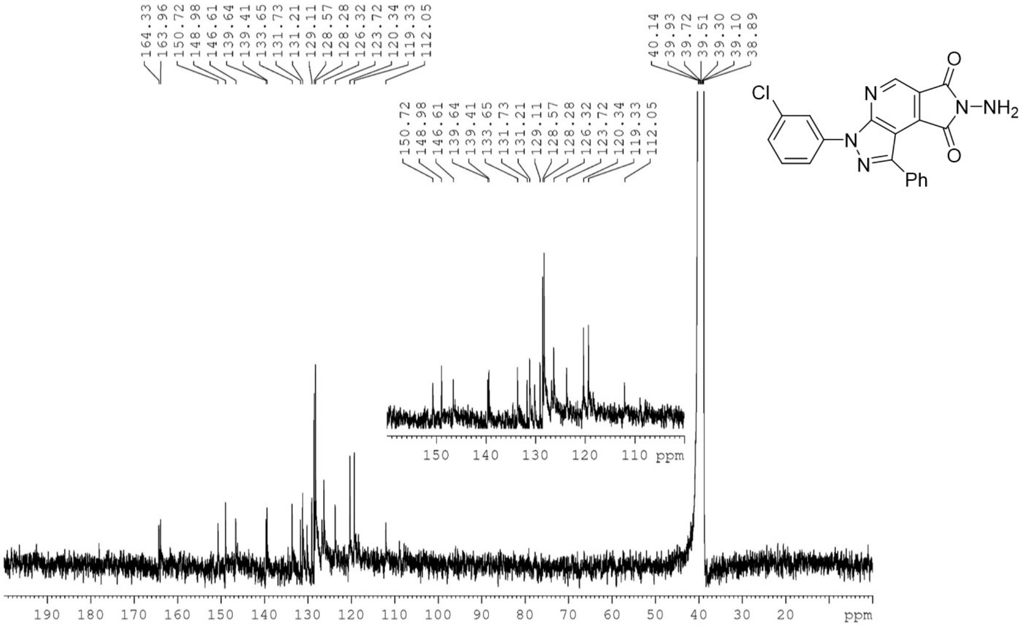

7d

7-Amino-3-(4-chlorophenyl)-1-phenylpyrazolo[3,4-*b*]pyrrolo[3,4-*d*] pyridine-6,8(3*H*,7*H*)-dione

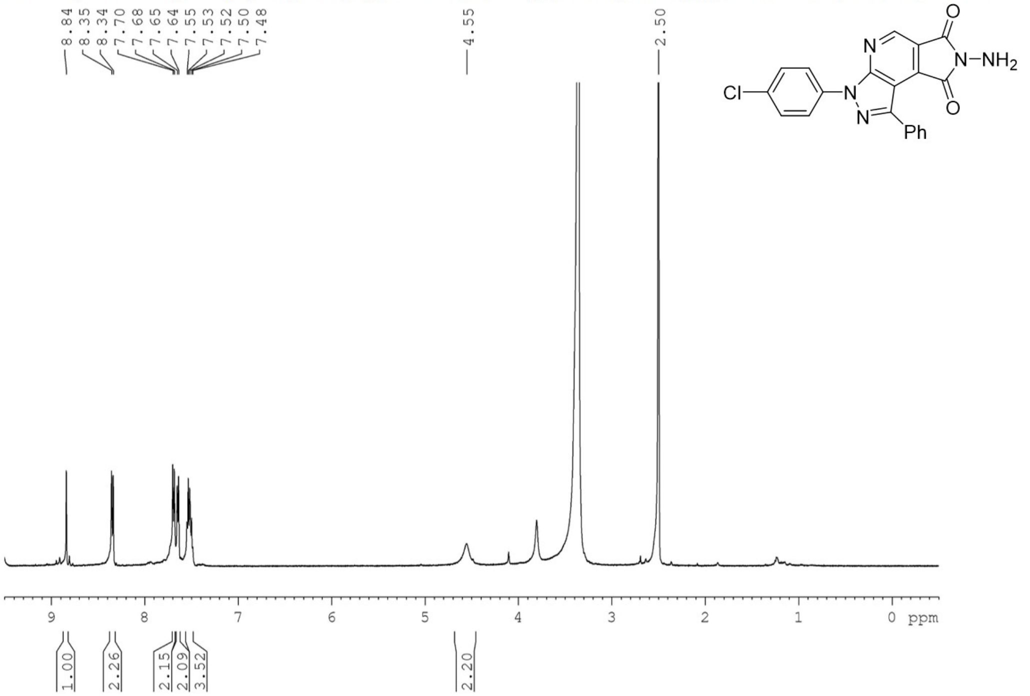

7d

7-Amino-3-(4-chlorophenyl)-1-phenylpyrazolo[3,4-*b*]pyrrolo[3,4-*d*] pyridine-6,8(3*H*,7*H*)-dione

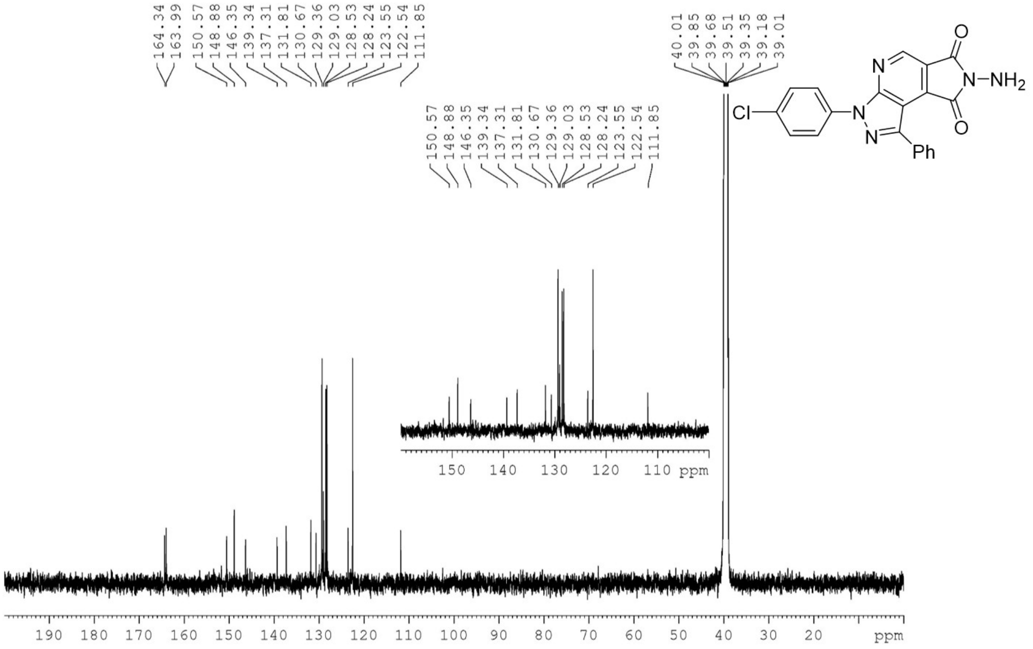

7e

7-Amino-3-(4-bromophenyl)-1-phenylpyrazolo[3,4-*b*]pyrrolo[3,4-*d*] pyridine-6,8(3*H*,7*H*)-dione

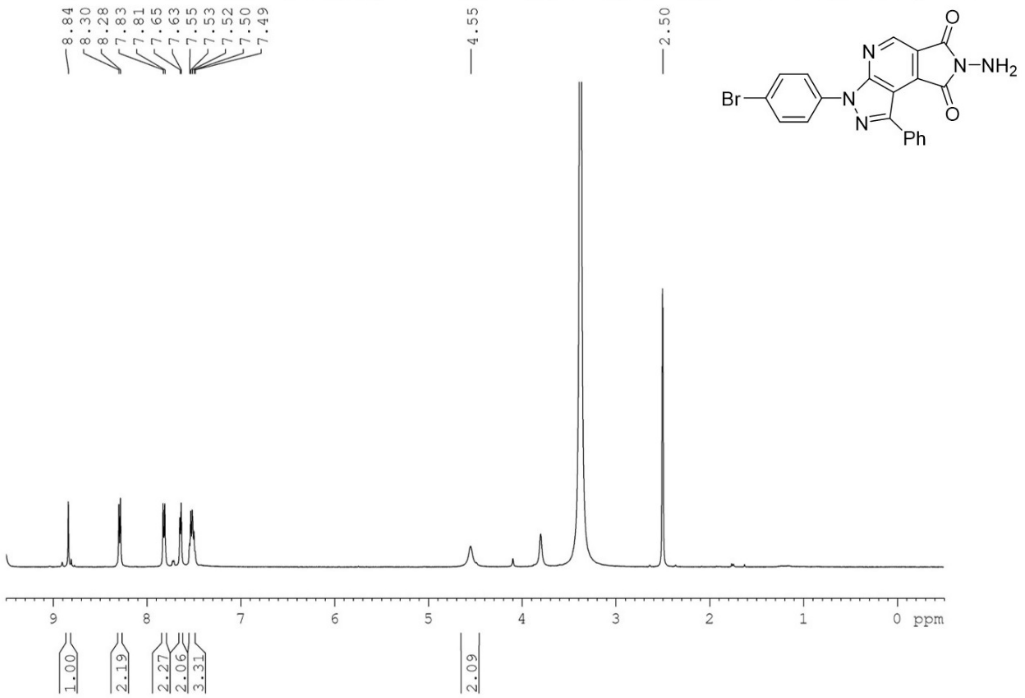

7e

7-Amino-3-(4-bromophenyl)-1-phenylpyrazolo[3,4-*b*]pyrrolo[3,4-*d*] pyridine-6,8(3*H*,7*H*)-dione

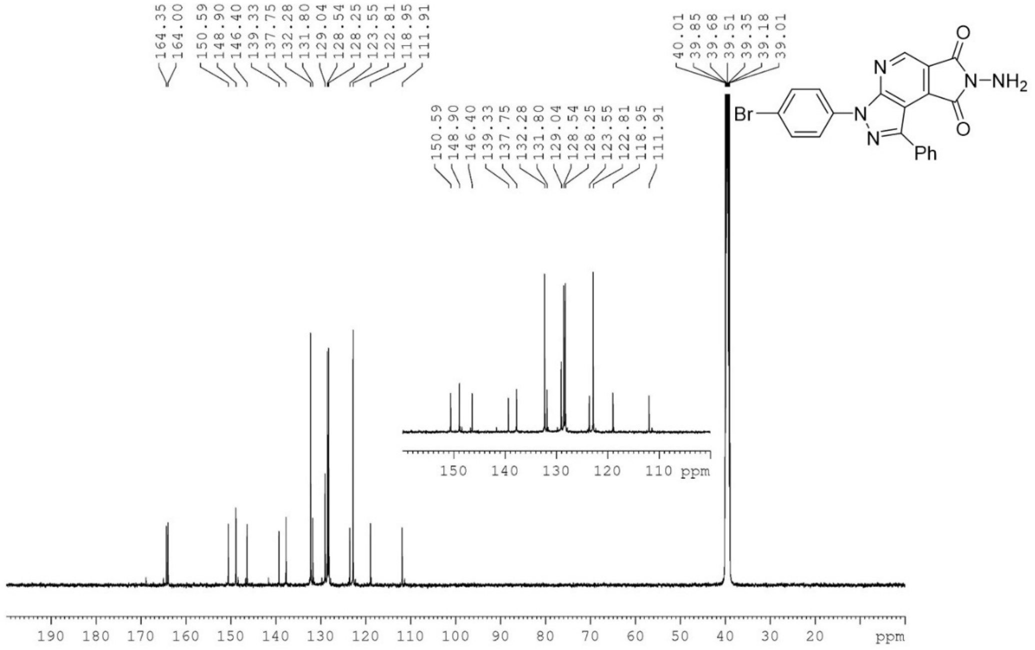

7f

7-Amino-1-phenyl-3-(*p*-tolyl)pyrazolo[3,4-*b*]pyrrolo[3,4-*d*] pyridine-6,8(3*H*,7*H*)-dione

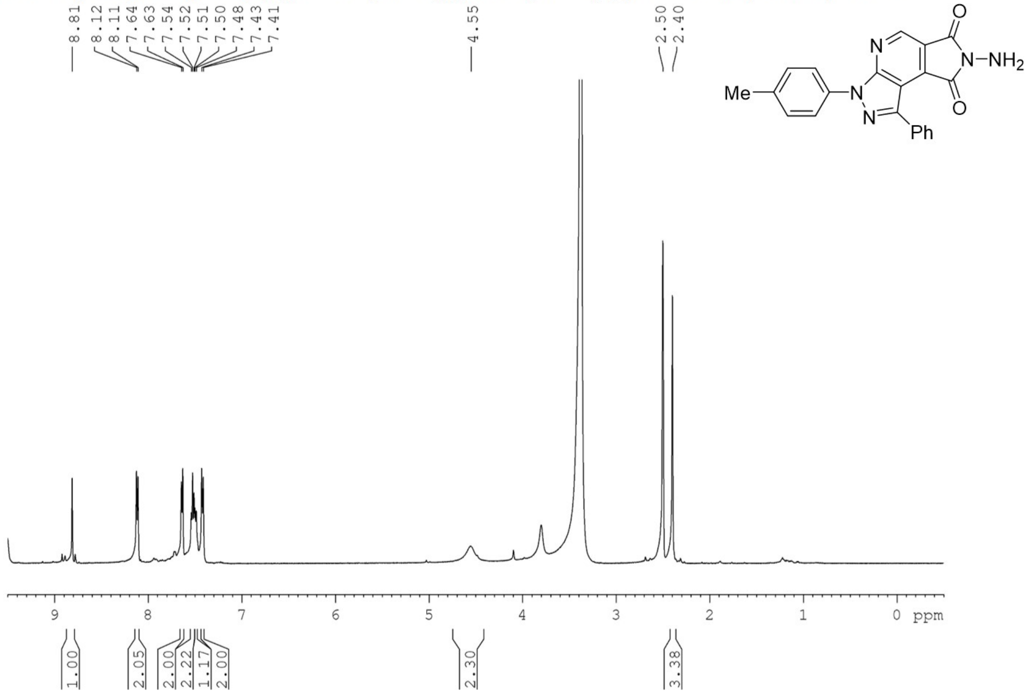

7f

7-Amino-1-phenyl-3-(*p*-tolyl)pyrazolo[3,4-*b*]pyrrolo[3,4-*d*] pyridine-6,8(3*H*,7*H*)-dione

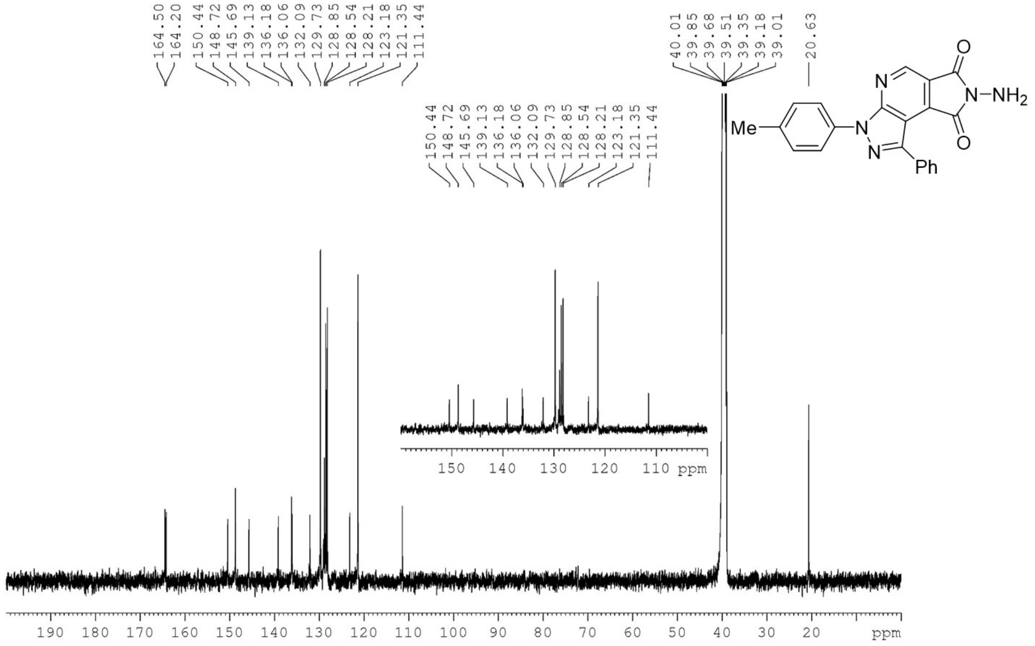

7g

7-Amino-3-(4-methoxyphenyl)-1-phenylpyrazolo[3,4-*b*]pyrrolo[3,4-*d*]pyridine-6,8-(3*H*,7*H*)-dione

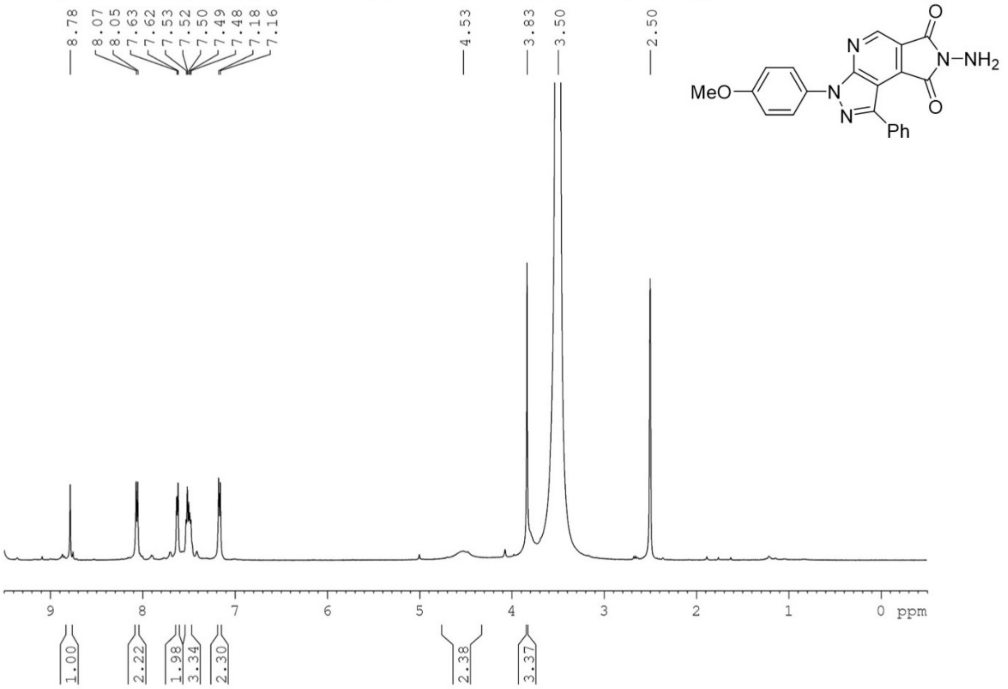

7g

7-Amino-3-(4-methoxyphenyl)-1-phenylpyrazolo[3,4-*b*]pyrrolo[3,4-*d*]pyridine-6,8-(3*H*,7*H*)-dione

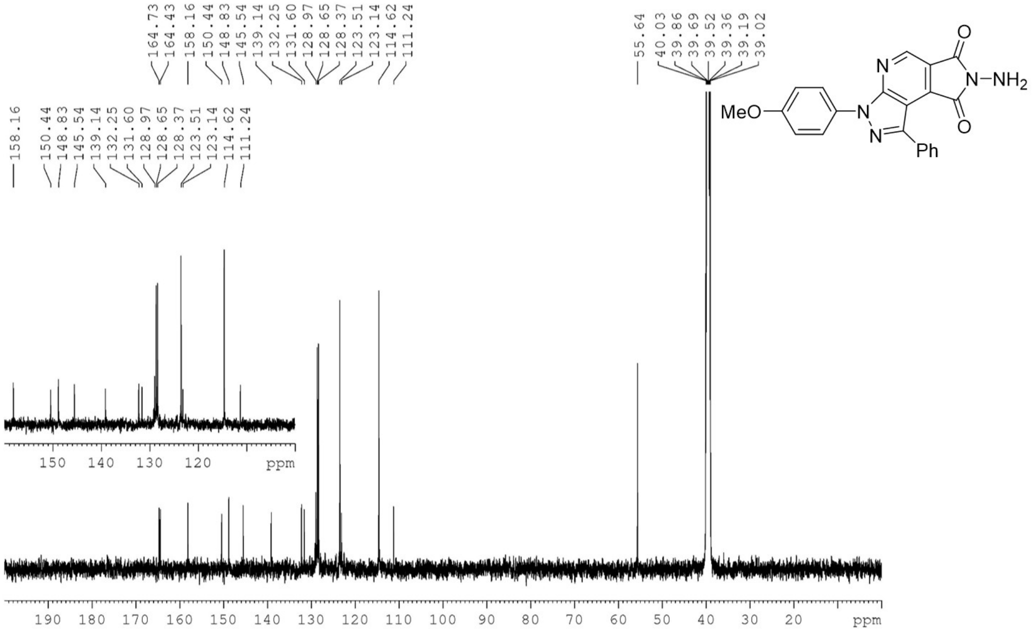

7h

7-Amino-3-(4-cyanophenyl)-1-phenylpyrazolo[3,4-*b*]pyrrolo[3,4-*d*]pyridine-6,8-(3*H*,7*H*)-dione

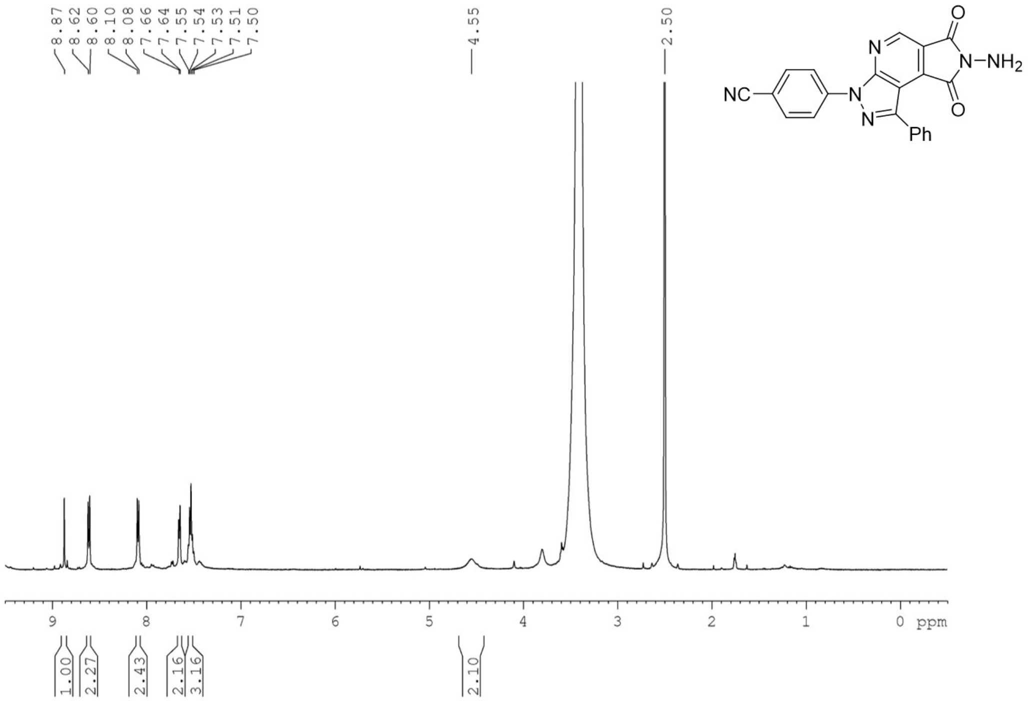

7h

7-Amino-3-(4-cyanophenyl)-1-phenylpyrazolo[3,4-*b*]pyrrolo[3,4-*d*]pyridine-6,8-(3*H*,7*H*)-dione

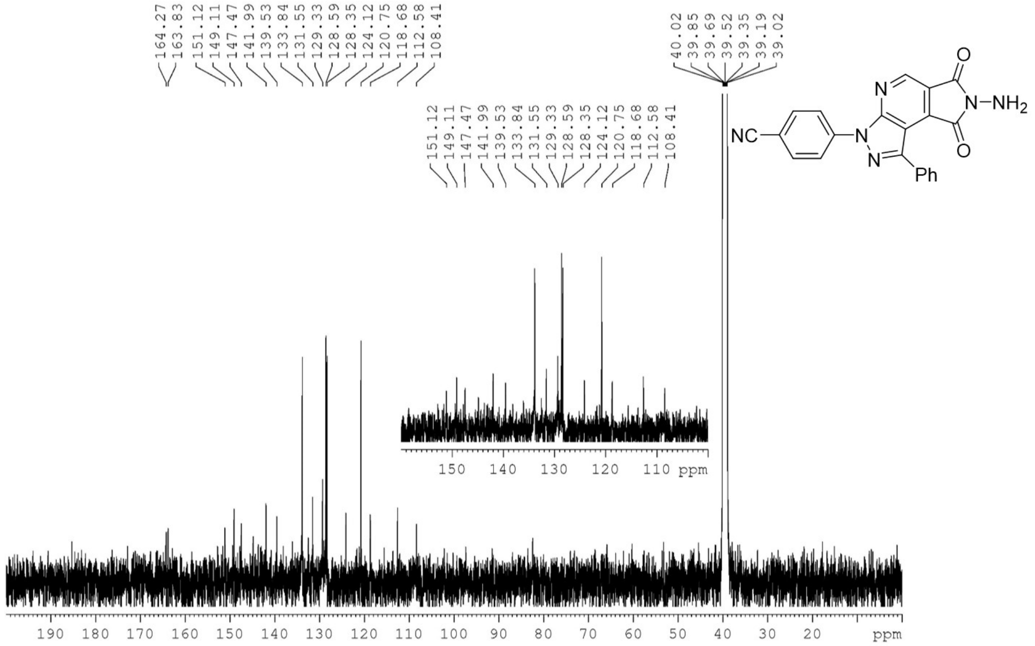

7i

7-Amino-3-(4-nitrophenyl)-1-phenylpyrazolo[3,4-*b*]pyrrolo[3,4-*d*]pyridine-6,8-(3*H*,7*H*)-dione

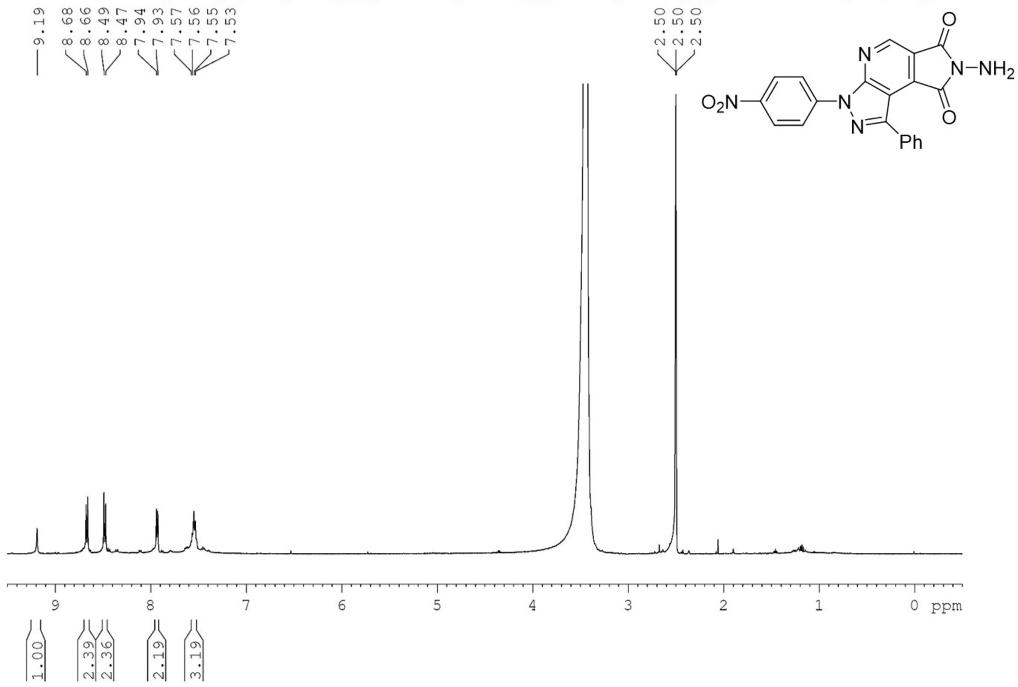

7i

7-Amino-3-(4-nitrophenyl)-1-phenylpyrazolo[3,4-*b*]pyrrolo[3,4-*d*]pyridine-6,8-(3*H*,7*H*)-dione

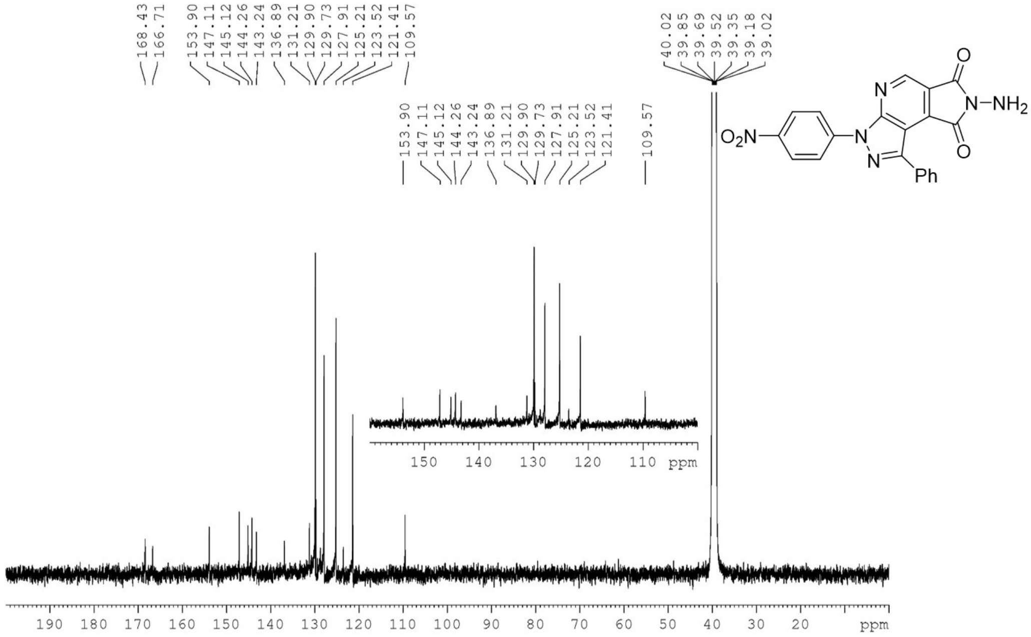

Supplement: Supplementary file 1 [file molecules-25-02409-s001.pdf]
